# Supplementary material for: ‘I gotta Feeling’: Exploring the effects of a smartphone app (Feelee) to enhance adolescents’ emotion regulation in forensic outpatient settings: A multiple single-case experimental design
Source: PLoS One. 2026 Feb 6;21(2):e0332111. doi: 10.1371/journal.pone.0332111 (PMC12880710; doi:10.1371/journal.pone.0332111)
Supplement: S1 File — (DOCX) [file pone.0332111.s001.docx]

**Appendix 1 –** Interview Topic Lists

[Topic list adolescents – follow-up measurement (Tf1) 1](#_Toc215135585)

[Topic list clinicians – follow-up measurement (Tf1) 3](#_Toc215135586)

[Topic list adolescents – 3-months follow-up measurement (Tf2) 5](#_Toc215135587)

## **Topic list adolescents – follow-up measurement (Tf1)**

**Opening questions**

1. What sparked your interest in joining this study?
2. What appealed to you about the Feelee app before you started?
3. Was the study as you expected?
4. Was the Feelee app what you expected it to be?

**General**

1. How did you experience using the Feelee app?
2. What did you find most noticeable about the app?
3. Were there things you liked less about using the Feelee app?

**Evaluation of Feelee**

1. Would you want to keep using Feelee alongside your treatment? Can you explain why?
2. Did you find it easy to fill in your emotions?
3. … knowing that you would discuss them with your clinician afterwards?
4. Did you always feel comfortable filling it in, or did you sometimes enter something different from how you actually felt?
5. How was it to report your emotions using emoji’s?
6. How did the daily check-ins with emoji’s go for you, and how did you like them?
7. What did you think of the movement and sleep data? What did you take from those insights during treatment?
8. Did using the Feelee app bring up topics in your treatment that you might not have discussed with your clinician otherwise?

**Emotions**

1. Has the Feelee app changed the way you look at your emotions?
2. Do you feel you have come to understand your emotions better?
3. Did the Feelee app help you put your emotions into words? Can you explain?
4. Were you always able to find the right emoji’s that matched how you felt?

**Motivation for treatment**

1. Did you experience the Feelee app as a helpful addition to your treatment? Why or how?
2. Did using the Feelee app make you feel differently about going to your weekly appointment with your clinician?
3. Do you feel that it helped speed up your treatment? How do you see that?

**Therapeutic relationship**

1. How was it for you to share the data from the Feelee app with your clinician (sensitive question)?
2. How did that feel for you?
3. Do you think the Feelee app contributed to feeling more understood by your clinician?
4. In what way, or why not?

**Future of Feelee**

1. What would you like to improve about the Feelee app? Do you have suggestions?
2. In what way would you like to use Feelee in your treatment? What would help you or be pleasant for you?
3. Would you recommend it to someone else, for example a friend or family member?

**Closing**

1. Is there anything else you would like to share with us?

## **Topic list clinicians – follow-up measurement (Tf1)**

**Opening questions**

1. What did you think of the study?
2. What did you think of the Feelee app?
3. Was it as you expected?

**General**

1. What was it like to use the Feelee app in your treatment sessions?
2. What stands out to you most from the sessions in which you discussed the Feelee data with the young person?
3. What was the added value of Feelee for you as a clinician?

**Evaluation**

1. How was it for you to discuss daily-life data from the client during treatment?
2. What did the Feelee app provide or contribute for you as a clinician during the treatment?
3. In what ways did the Feelee app support the treatment?
4. Where do you see the main added value of the Feelee app?
   1. Added value for the young person’s emotion regulation (recognizing, feeling, understanding or gaining insight into their emotions).
   2. In your own words, can you explain what kind of change Feelee brought to the treatment? Where, in your view, lies the core contribution?

**Emotions**

1. Did the Feelee app support your client in recognizing and sensing their emotions?
2. Do you think the Feelee app helped your client understand their emotions better?
3. Did the Feelee app make it easier for your client to talk about their emotions during the sessions?
4. Did Feelee contribute to your client’s ability to reflect?

**Motivation**

1. Did you experience the Feelee app as a helpful addition to the treatment? Why or how?
2. Did Feelee contribute to increasing young people’s engagement in treatment?
3. Did Feelee help motivate the client for treatment?

**Therapeutic relationship**

1. Do you feel the client felt more understood during the treatment?
2. Was the Feelee app helpful for you as a clinician to better understand the client?
3. Did Feelee contribute to the therapeutic relationship?

**Future use of Feelee**

1. Would you like to continue using the Feelee app in treatment?
2. For which clients or types of client needs do you think Feelee is most suitable?
3. What adjustments or improvements would you like to see in the Feelee app, particularly from the perspective of supporting your work as a clinician?
4. Do you have suggestions for the further implementation of the Feelee app in the future?

**Closing**

1. Is there anything else you would like to share?
2. About the study or about the Feelee app?
3. Do you have any tips for the research team?
4. Is there anything we should keep in mind?
5. Do you have suggestions for improving the study?

## **Topic list adolescents – 3-months follow-up measurement (Tf2)**

This second interview focuses on longer-term effects. How does the young person look back on using the Feelee app, three months later?

**Opening questions**

1. How are you doing?
2. Looking back after three months, how do you feel about using the Feelee app?
3. And what about the study? How did you experience that?
4. Could you share any strengths and points for improvement?

**Feelee in treatment**

1. Are you currently using the Feelee app alongside your treatment?
2. How do you use the Feelee app in your treatment at the moment?

**Evaluation**

1. What has the Feelee app meant for you within your treatment?
2. Has the Feelee app been helpful for you in any way over the past few months?
3. Looking back on the study, has the Feelee app changed anything for you in your daily life? Could you explain?

**Emotions and habits**

1. How are things going for you when it comes to understanding your emotions?
2. Do you feel that something in your life (or in your body) has changed recently?
3. Can you describe that?
4. Do you feel that you deal with your emotions differently now?
5. Have you made changes in your daily life because of using the Feelee app, for example based on your movement or sleep data?

**Treatment (motivation and therapeutic relationship)**

1. How is your treatment going at the moment?
2. Have things changed for you recently, for example at school, in your family, with friends or with your health? And do you think any of this is related to using the Feelee app?

**Closing**

1. Is there anything else you would like to share with us?
2. Do you have a wish for the future, for yourself or for the Feelee app?
