# Supplementary material for: ‘I gotta Feeling’: Exploring the effects of a smartphone app (Feelee) to enhance adolescents’ emotion regulation in forensic outpatient settings: A multiple single-case experimental design
Source: PLoS One. 2026 Feb 6;21(2):e0332111. doi: 10.1371/journal.pone.0332111 (PMC12880710; doi:10.1371/journal.pone.0332111)
Supplement: S2 File — (DOCX) [file pone.0332111.s002.docx]

**Appendix 2 –** Overview results participants

**Figure 1. Overview participants’ study progress**

| **Participant** | **Age** | **Sex** | **T0** | **Baseline** | | **Intervention** | | | | | | **T1** | **Follow-up** | | **T2** | **T3** | **% DQ done** |
| --- | --- | --- | --- | --- | --- | --- | --- | --- | --- | --- | --- | --- | --- | --- | --- | --- | --- |
|  |  |  |  | Daily questionnaire | | Feelee in treatment | | | | Daily questionnaire | |  | Daily questionnaire | |  |  |  |
|  |  |  |  | Done | Missing | 1 | 2 | 3 | 4 | Done | Missing |  | Done | Missing |  |  |  |
| **1** | 18 | Girl | ✓ | 14 | 0 | - | ✓ | ✓ | ✓ | 24 | 8 | ✓ | 8 | 4 | ✓ | ✓ | 79% |
| **2** | 20 | Boy | ✓ | 7 | 7 | D/O | - | - | - | - | - | - | - | - | - | - | 50% |
| **3** | 21 | Boy | ✓ | 13 | 2 | - | - | - | D/O | 17 | 11 | - | - | - | - | - | 70% |
| **4** | 15 | Boy | ✓ | 2 | 12 | ✓ | - | - | - | 1 | 27 | ✓ | 2 | 14 | D/O | - | 9% |
| **5** | 17 | Boy | ✓ | 8 | 13 | ✓ | ✓ | ✓ | N/A | 4 | 31 | ✓ | 4 | 11 | ✓ | - | 23% |
| **6** | 15 | Boy | ✓ | 8 | 6 | N/A | D/O | - | - | 0 | 16 | - | - | - | - | - | 27% |
| **7** | 19 | Boy | ✓ | 10 | 8 | N/A | D/O | - | - | 6 | 1 | - | - | - | - | - | 64% |
| **8** | 19 | Girl | ✓ | 8 | 7 | ✓ | ✓ | ✓ | ✓ | 17 | 11 | ✓ | 8 | 6 | ✓ | ✓ | 58% |
| **9** | 16 | Boy | ✓ | 8 | 6 | ✓ | - | ✓ | - | 15 | 16 | - | 8 | 13 | ✓ | - | 47% |
| **10** | 16 | Boy | ✓ | 14 | 0 | ✓ | - | - | - | 35 | 0 | ✓ | 4 | 12 | ✓ | ✓ | 82% |
| **11** | 17 | Boy | ✓ | 4 | 10 | D/O | - | - | - | - | - | - | - | - | - | - | 29% |
| **12** | 16 | Boy | ✓ | 14 | 0 | ✓ | - | - | - | 24 | 19 | ✓ | 10 | 4 | ✓ | - | 68% |
| **13** | 17 | Girl | ✓ | 4 | 11 | - | N/A | D/O | - | 1 | 20 | - | - | - | - | - | 14% |
| **14** | 20 | Boy | ✓ | 15 | 0 | ✓ | N/A | N/A | N/A | 31 | 15 | ✓ | 6 | 15 | ✓ | - | 63% |
| **15** | 18 | Boy | ✓ | 19 | 1 | ✓ | ✓ | ✓ | N/A | 34 | 1 | ✓ | 22 | 6 | ✓ | - | 90% |
| **16** | 15 | Boy | ✓ | 15 | 0 | - | N/A | N/A | N/A | 30 | 18 | ✓ | 9 | 14 | ✓ | - | 63% |
| **17** | 18 | Boy | ✓ | 15 | 1 | N/A | - | ✓ | N/A | 24 | 4 | ✓ | 20 | 7 | ✓ | - | 83% |
| **18** | 23 | Boy | ✓ | 14 | 0 | ✓ | N/A | N/A | N/A | 25 | 10 | ✓ | 6 | 16 | ✓ | ✓ | 63% |
| **19** | 17 | Boy | ✓ | 28 | 0 | ✓ | N/A | N/A | N/A | 35 | 0 | ✓ | 14 | 0 | ✓ | ✓ | 100% |
| **20** | 19 | Boy | ✓ | 6 | 13 | N/A | N/A | N/A | N/A | 5 | 23 | ✓ | 9 | 27 | ✓ | - | 24% |
| **21** | 17 | Boy | ✓ | 8 | 10 | - | D/O | - | - | - | - | - | - | - | - | - | 44% |
| **22** | 18 | Boy | ✓ | 15 | 2 | ✓ | ✓ | N/A | N/A | 9 | 26 | ✓ | 5 | 16 | ✓ | - | 40% |

Note. D/O – drop-out of study, N/A – no data applicable from clinician.

**Table 1-3.** All individual scores recognize (clarity) item

**Baseline – Intervention Intervention – follow-up Baseline – follow-up**

| **pp** | **Cohens’ d** | **p-value** | **TAU-U** | **p-value TAU-U** |
| --- | --- | --- | --- | --- |
| **1** | -1.063 | 0.010* | -0.47 | 0.111 |
| **3** | 0 |  | 1 | 0.999 |
| **8** | -1.146 | 0.003* | -0.49 | 0.056 |
| **9** | -0.974 | 0.498 | -0.44 | 0.975 |
| **10** | -0.297 | 0.02* | -0.22 | 0.071 |
| **12** | -0.086 | 0.50 | -0.04 | 0.991 |
| **14** | 0.788 | 0.493 | 0.3 | 0.922 |
| **15** | 0.206 | 0.50 | 0.1 | 0.999 |
| **16** | 0 |  | 1 | 0.999 |
| **17** | -1.02 | 0.050* | -0.43 | 0.106 |
| **18** | 0.573 | 0.786 | 0.3 | 0.999 |
| **19** | -0.054 | 0.563 | 0 | 0.783 |
| **22** | 0.344 | 0.861 | 017 | 0.942 |

| **pp** | **Cohens’ d** | **p-value** | **TAU-U** | **p-value TAU-U** |
| --- | --- | --- | --- | --- |
| **1** | 1.242 | 0.515 | 0.47 | 0.009* |
| **8** | -0.393 | 0.004* | -0.18 | 0.38 |
| **9** | -0.329 | 0.497 | -0.12 | 0.422 |
| **10** | 0 | 0.764 | 0.33 | 0.016* |
| **12** | -0.168 | 0.50 | -0.08 | 0.685 |
| **14** | 0.567 | 0.480 | 0.23 | 0.18 |
| **15** | -0.226 |  | -0.11 | 0.425 |
| **16** | 0 |  | 1 | 1 |
| **17** | 0.445 | 0.470 | 0.23 | 0.12 |
| **18** | -0.323 | 0.977 | -0.08 | 0.653 |
| **19** | 0.583 | 0.829 | 0.2 | 0.072 |
| **22** | -0.255 | 0.523 | -0.13 | 0.672 |

| **pp** | **Cohens’ d** | **p-value** | **TAU-U** | **p-value TAU-U** |
| --- | --- | --- | --- | --- |
| **1** | -0.104 | 0.490 | -0.02 | 0.97 |
| **8** | -1.443 | 0.006* | -0.61 | 0.018* |
| **9** | -0.861 | 0.498 | -0.39 | 0.026* |
| **10** | -0.029 | 0.772 | 0.02 | 0.937 |
| **12** | -0.187 | 0.50 | -0.09 | 0.684 |
| **14** | 0.916 | 0.480 | 0.38 | 0.083 |
| **15** | 0 |  | 1 | 1 |
| **16** | 0 |  | 1 | 1 |
| **17** | -0.613 | 0.463 | -0.28 | 0.105 |
| **18** | 0.289 | 0.973 | 0.27 | 0.256 |
| **19** | 0.446 | 0.843 | 0.21 | 0.12 |
| **22** | 0.062 | 0.524 | 0.01 | 1 |

***Note.*** Cohen’s d effect size: d = .01 - very small; d = .20 – small; d = .50 - medium; d = 0.80 – large; d = 1.20 – very large; d = 2.00 – huge. p-value is significant at < 0.05 (marked with *). TAU-U: TAU-U= < 0.20 – weak, TAU-U = <0.60 – moderate, TAU-U = <.80 – strong, TAU-U = <1.00 – very strong.

**Table 4-6.** All individual scores suppression item

**Baseline – Intervention Intervention – follow-up Baseline – follow-up**

| **pp** | **Cohens’ d** | **p-value** | **TAU-U** | **p-value TAU-U** |
| --- | --- | --- | --- | --- |
| **1** | -0.043 | 0.337 | 0.01 | 0.939 |
| **3** | 1.351 | 0.929 | -0.53 | 0.00* |
| **8** | 1.185 | 0.981 | -0.45 | 0.009* |
| **9** | -0.261 | 0.001* | 0.33 | 0.015* |
| **10** | 0.033 | 0.539 | -0.03 | 0.842 |
| **12** | -0.4 | 0.596 | 0.11 | 0.48 |
| **14** | 0.238 | 0.878 | -0.09 | 0.531 |
| **15** | -0.27 | 0.50 | -0.1 | 0.514 |
| **16** | 1.344 | 0.998 | -0.44 | 0.002* |
| **17** | 2.145 | 0.925 | -0.64 | 0 |
| **18** | 1.635 | >0.999 | -0.62 | 0 |
| **19** | 0.109 | 0.522 | -0.08 | 0.436 |
| **22** | 0.375 | 0.888 | -0.23 | 0.234 |

| **pp** | **Cohens’ d** | **p-value** | **TAU-U** | **p-value TAU-U** |
| --- | --- | --- | --- | --- |
| **1** | 0.059 | 0.537 | -0.01 | 0.945 |
| **8** | -0.526 | 0.967 | 0.3 | 0.097 |
| **9** | 0.232 | 0.003* | -0.03 | 0.836 |
| **10** | 0.59 | 0.945 | -0.29 | 0.033 |
| **12** | 0.426 | 0.494 | -0.15 | 0.392 |
| **14** | -0.182 | 0.826 | 0.05 | 0.744 |
| **15** | 0 | 0.50 | 0 | 1 |
| **16** | 0.213 | 0.915 | -0.04 | 0.809 |
| **17** | -0.024 | 0.901 | 0 | 1 |
| **18** | 0.045 | >0.999 | 0 | 1 |
| **19** | 0.223 | 0.738 | -0.05 | 0.637 |
| **22** | -0.641 | 0.455 | 0.48 | 0.069 |

| **pp** | **Cohens’ d** | **p-value** | **TAU-U** | **p-value TAU-U** |
| --- | --- | --- | --- | --- |
| **1** | 0.025 | 0.513 | -0,03 | 0.89 |
| **8** | 0.66 | 0.968 | -0,3 | 0.187 |
| **9** | 0.02 | 0.003* | 0.23 | 0.16 |
| **10** | 0.577 | 0.945 | -0.33 | 0.073 |
| **12** | -0.162 | 0.487 | -0.01 | 1 |
| **14** | 0.058 | 0.833 | -0.02 | 0.965 |
| **15** | -0.238 | 0.50 | -0.09 | 0.598 |
| **16** | 1.087 | 0.911 | -0.43 | 0.039* |
| **17** | 2.109 | 0.895 | -0.66 | 0 |
| **18** | 1.967 | >0.999 | -0.71 | 0.001* |
| **19** | 0.287 | 0.739 | -0.09 | 0.515 |
| **22** | -0.258 | 0.455 | 0.13 | 0.561 |

***Note.*** Cohen’s d effect size: d = .01 - very small; d = .20 – small; d = .50 - medium; d = 0.80 – large; d = 1.20 – very large; d = 2.00 – huge. p-value is significant at < 0.05 (marked with *). TAU-U: TAU-U= < 0.20 – weak, TAU-U = <0.60 – moderate, TAU-U = <.80 – strong, TAU-U = <1.00 – very strong.

**Table 7-9.** All individual scores reflection, comprehension (rumination) item

**Baseline – Intervention Intervention – follow-up Baseline – follow-up**

| **pp** | **Cohens’ d** | **p-value** | **TAU-U** | **p-value TAU-U** |
| --- | --- | --- | --- | --- |
| **1** | 0.319 | 0.541 | -0.09 | 0.566 |
| **3** | 2.13 | 0.963 | -0.66 | 0 |
| **8** | 1.734 | 0.997 | -0.56 | 0.0001* |
| **9** | 0.169 | 0.439 | -0.07 | 0.632 |
| **10** | 0.982 | 0.986 | -0.35 | 0.006* |
| **12** | -0.18 | 0.676 | 0.04 | 0.81 |
| **14** | 0.311 | 0.989 | -0.25 | 0.086 |
| **15** | -0.399 | 0.50 | -0.03 | 0.845 |
| **16** | 1.911 | 0.998 | -0.55 | 0 |
| **17** | 0.909 | 0.999 | -0.35 | 0.017* |
| **18** | 2.054 | >0.999 | -0.68 | 0 |
| **19** | -0.327 | 0.435 | 0.08 | 0.45 |
| **22** | 0.367 | 0.417 | -0.18 | 0.366 |

| **pp** | **Cohens’ d** | **p-value** | **TAU-U** | **p-value TAU-U** |
| --- | --- | --- | --- | --- |
| **1** | 0.224 | 0.845 | -0.03 | 0.842 |
| **8** | -0.002 | 0.991 | 0.05 | 0.815 |
| **9** | 0.049 | 0.493 | 0.07 | 0.649 |
| **10** | 0.81 | 0.998 | -0.29 | 0.027* |
| **12** | 0.754 | 0.868 | -0.3 | 0.074 |
| **14** | 0.881 | 0.983 | -0.32 | 0.042 |
| **15** | -0.391 | 0.50 | 0.19 | 0.162 |
| **16** | -0.612 | 0.956 | 0.02 | 0.934 |
| **17** | 0.321 | 0.996 | -0.11 | 0.424 |
| **18** | -0.274 | >0.999 | 0.08 | 0.645 |
| **19** | -0.367 | 0.095 | 0.17 | 0.15 |
| **22** | -1.39 | 0.006* | 0.44 | 0.088 |

| **pp** | **Cohens’ d** | **p-value** | **TAU-U** | **p-value TAU-U** |
| --- | --- | --- | --- | --- |
| **1** | 0.319 | 0.857 | -0.13 | 0.53 |
| **8** | 2.13 | 0.990 | -0.72 | 0.002* |
| **9** | 1.734 | 0.472 | -0.06 | 0.721 |
| **10** | -0.5 | 0.999 | -0.69 | 0 |
| **12** | 0.982 | 0.858 | -0.25 | 0.187 |
| **14** | 2.054 | 0.978 | -0.62 | 0.004* |
| **15** | 0.169 | 0.50 | 0.05 | 0.772 |
| **16** | -0.18 | 0.959 | -0.39 | 0.052 |
| **17** | -0.399 | 0.996 | -0.55 | 0 |
| **18** | 1.911 | >0.999 | -0.72 | 0.001* |
| **19** | -0.274 | 0.102 | 0.16 | 0.237 |
| **22** | 0.311 | 0.001* | 0.27 | 0.185 |

***Note****.* Cohen’s d effect size: d = .01 - very small; d = .20 – small; d = .50 - medium; d = 0.80 – large; d = 1.20 – very large; d = 2.00 – huge. p-value is significant at < 0.05 (marked with *). TAU-U: TAU-U= < 0.20 – weak, TAU-U = <0.60 – moderate, TAU-U = <.80 – strong, TAU-U = <1.00 – very strong.

**Table 10-12.** All individual scores reflection, comprehension (reappraisal) item

**Baseline – Intervention Intervention – follow-up Baseline – follow-up**

| **pp** | **Cohens’ d** | **p-value** | **TAU-U** | **p-value TAU-U** |
| --- | --- | --- | --- | --- |
| **1** | 0.047 | 0.658 | -0.06 | 0.712 |
| **3** | 1.297 | 0.954 | -0.46 | 0.011* |
| **8** | 1.884 | 0.999 | -0.06 | 0.0012* |
| **9** | 0.676 | 0.726 | -0.2 | 0.124 |
| **10** | 0.613 | 0.930 | -0.23 | 0.077 |
| **12** | -0.753 | 0.034* | 0.28 | 0.067 |
| **14** | 0.012 | 0.436 | -0.05 | 0.705 |
| **15** | -0.114 | 0.50 | 0.06 | 0.718 |
| **16** | 1.406 | 0.934 | -0.47 | 0.001* |
| **17** | 0.817 | 0.991 | -0.34 | 0.021* |
| **18** | -0.549 | 0.409 | 0.12 | 0.421 |
| **19** | 0.615 | 0.972 | -0.23 | 0.015* |
| **22** | -0.271 | 0.151 | 0.21 | 0.274 |

| **pp** | **Cohens’ d** | **p-value** | **TAU-U** | **p-value TAU-U** |
| --- | --- | --- | --- | --- |
| **1** | 0.599 | 0.993 | -0.19 | 0.238 |
| **8** | -0.457 | 0.994 | 0.26 | 0.155 |
| **9** | 0.642 | 0.998 | -0.19 | 0.164 |
| **10** | 0.781 | 0.992 | -0.3 | 0.07 |
| **12** | 0.167 | 0.999 | -0.04 | 0.762 |
| **14** | 0.415 | 0.778 | -0.12 | 0.43 |
| **15** | 0.049 | 0.50 | -0.02 | 0.881 |
| **16** | 0.737 | 0.838 | -0.3 | 0.067 |
| **17** | 0.571 | 0.890 | -0.19 | 0.162 |
| **18** | -0.72 | <0.001* | 0.17 | 0.302 |
| **19** | 0.826 | 0.909 | -0.31 | 0.004* |
| **22** | -0.497 | 0.013* | 0.22 | 0.411 |

| **pp** | **Cohens’ d** | **p-value** | **TAU-U** | **p-value TAU-U** |
| --- | --- | --- | --- | --- |
| **1** | 0.047 | 0.989 | -0.39 | 0.044* |
| **8** | 1.297 | 0.996 | -0.62 | 0.007* |
| **9** | 0.613 | 0.998 | -0.24 | 0.148 |
| **10** | 1.884 | 0.992 | -0.46 | 0.011* |
| **12** | 0.676 | 0.508 | -0.03 | 0.91 |
| **14** | -0.072 | 0.774 | -0.21 | 0.338 |
| **15** | -0.753 | 0.50 | 0.05 | 0.794 |
| **16** | -0.114 | 0.837 | -0.62 | 0.003* |
| **17** | 1.406 | 0.886 | -0.66 | 0 |
| **18** | 0.817 | <0.001* | 0.38 | 0.093 |
| **19** | 0.012 | 0.913 | -0.53 | 0 |
| **22** | 0.615 | 0.012* | 0.32 | 0.12 |

####

***Note.*** Cohen’s d effect size: d = .01 - very small; d = .20 – small; d = .50 - medium; d = 0.80 – large; d = 1.20 – very large; d = 2.00 – huge. p-value is significant at < 0.05 (marked with *). TAU-U: TAU-U= < 0.20 – weak, TAU-U = <0.60 – moderate, TAU-U = <.80 – strong, TAU-U = <1.00 – very strong.

**Table 13-15.** All individual scores manage (impulse) item

**Baseline – Intervention Intervention – follow-up Baseline – follow-up**

| **pp** | **Cohens’ d** | **p-value** | **TAU-U** | **p-value TAU-U** |
| --- | --- | --- | --- | --- |
| **1** | -0.928 | 0.491 | -0.36 | 0.023* |
| **3** | 0.323 | 0.50 | 0.16 | 0.451 |
| **8** | -1.546 | 0.989 | -0.58 | 0.002* |
| **9** | -0.159 | 0.571 | -0.16 | 0.264 |
| **10** | -0.483 | 0.916 | -0.19 | 0.149 |
| **12** | 0.418 | 0.509 | 0.21 | 0.171 |
| **14** | 0.794 | 0.084 | 0.33 | 0.025* |
| **15** | 0 |  | 1 | 1 |
| **16** | 0.707 | 0.50 | 0.32 | 0.039* |
| **17** | -0.69 | 0.572 | -0.29 | 0.064 |
| **18** | 0.578 | 0.117 | 0.28 | 0.058 |
| **19** | 0.246 | 0.21 | 0.15 | 0.136 |
| **22** | 0.051 | 0.209 | 0.04 | 0.871 |

| **pp** | **Cohens’ d** | **p-value** | **TAU-U** | **p-value TAU-U** |
| --- | --- | --- | --- | --- |
| **1** | 1.214 | 0.249 | 0.49 | 0.006* |
| **8** | 0.567 | 0.851 | 0.2 | 0.322 |
| **9** | -0.866 | 0.888 | -0.43 | 0.004* |
| **10** | 0.868 | 0.232 | 0.3 | 0.026* |
| **12** | -0.241 | 0.496 | -0.11 | 0.561 |
| **14** | 0.86 | 0.150 | 0.32 | 0.053 |
| **15** | 0 |  | 1 | 1 |
| **16** | 0 | 0.50 | 1 | 1 |
| **17** | -0.304 | 0.983 | -0.11 | 0.451 |
| **18** | -0.479 | 0.577 | -0.19 | 0.253 |
| **19** | -1 | 0.008* | 0.37 | 0.001* |
| **22** | 0.28 | 0.144 | 0.15 | 0.617 |

| **pp** | **Cohens’ d** | **p-value** | **TAU-U** | **p-value TAU-U** |
| --- | --- | --- | --- | --- |
| **1** | -0.049 | 0.229 | 0.04 | 0.883 |
| **8** | -0.672 | 0.850 | -0.35 | 0.152 |
| **9** | -1.522 | 0.895 | -0.62 | 0 |
| **10** | 0.347 | 0.237 | 0.16 | 0.399 |
| **12** | 0.284 | 0.497 | 0.17 | 0.389 |
| **14** | 1.253 | 0.139 | 0.49 | 0.023* |
| **15** | 0 |  | 1 | 1 |
| **16** | 0.476 | 0.50 | 0.23 | 0.341 |
| **17** | -1.08 | 0.984 | -0.41 | 0.013* |
| **18** | 0.076 | 0.584 | -0.03 | 0.918 |
| **19** | 0.846 | 0.009* | 0.37 | 0.007* |
| **22** | 0.315 | 0.134 | 0.16 | 0.482 |

***Note****.* Cohen’s d effect size: d = .01 - very small; d = .20 – small; d = .50 - medium; d = 0.80 – large; d = 1.20 – very large; d = 2.00 – huge. p-value is significant at < 0.05 (marked with *). TAU-U: TAU-U= < 0.20 – weak, TAU-U = <0.60 – moderate, TAU-U = <.80 – strong, TAU-U = <1.00 – very strong.

**Table 16-18.** All individual scores manage (distraction) item

**Baseline – Intervention Intervention – follow-up Baseline – follow-up**

| **pp** | **Cohens’ d** | **p-value** | **TAU-U** | **p-value TAU-U** |
| --- | --- | --- | --- | --- |
| **1** | 0.045 | 0.176 | 0.06 | 0.677 |
| **3** | 1.322 | 0.979 | -0.52 | 0.004* |
| **8** | 1.344 | 0.953 | -0.4 | 0.022* |
| **9** | 0.783 | 0.516 | -0.26 | 0.05* |
| **10** | -0.177 | 0.509 | 0.12 | 0.408 |
| **12** | -0.246 | 0.377 | 0.22 | 0.153 |
| **14** | 0.89 | 0.994 | -0.35 | 0.011* |
| **15** | -0.153 | 0.50 | -0.19 | 0.192 |
| **16** | 1.218 | 0.858 | -0.36 | 0.012* |
| **17** | 1.402 | 0.999 | -0.49 | 0.001* |
| **18** | 1.315 | >0.999 | -0.51 | 0 |
| **19** | 0.535 | 0.939 | -0.22 | 0.024* |
| **22** | 0.164 | 0.557 | -0.08 | 0.695 |

| **pp** | **Cohens’ d** | **p-value** | **TAU-U** | **p-value TAU-U** |
| --- | --- | --- | --- | --- |
| **1** | 0.251 | 0.521 | -0.08 | 0.611 |
| **8** | -0.104 | 0.970 | 0.14 | 0.455 |
| **9** | 0.637 | 0.999 | -0.14 | 0.313 |
| **10** | -0.532 | 0.50 | 0.26 | 0.077 |
| **12** | 0.183 | 0.503 | -0.09 | 0.579 |
| **14** | 0.306 | 0.966 | -0.14 | 0.377 |
| **15** | -0.307 | 0.50 | 0.15 | 0.274 |
| **16** | -0.406 | 0.529 | 0.17 | 0.289 |
| **17** | 0.345 | 0.982 | -0.14 | 0.318 |
| **18** | 0.081 | >0.999 | -0.03 | 0.849 |
| **19** | -0.448 | 0.431 | 0.21 | 0.058 |
| **22** | -0.148 | 0.66 | 0.1 | 0.734 |

| **pp** | **Cohens’ d** | **p-value** | **TAU-U** | **p-value TAU-U** |
| --- | --- | --- | --- | --- |
| **1** | 0.371 | 0.523 | -0.07 | 0.729 |
| **8** | 1.389 | 0.976 | -0.41 | 0.072 |
| **9** | 1.553 | 0.999 | -0.37 | 0.026* |
| **10** | -0.795 | 0.50 | 0.43 | 0.027* |
| **12** | -0.08 | 0.504 | 0.1 | 0.615 |
| **14** | 1.494 | 0.967 | -0.55 | 0.01* |
| **15** | -0.238 | 0.50 | -0.09 | 0.598 |
| **16** | 0.737 | 0.525 | -0.21 | 0.312 |
| **17** | 1.956 | 0.981 | -0.6 | 0 |
| **18** | 1.752 | >0.999 | -0.64 | 0.004* |
| **19** | 0.109 | 0.428 | -0.05 | 0.712 |
| **22** | 0.058 | 0.652 | 0 | 1 |

***Note****.* Cohen’s d effect size: d = .01 - very small; d = .20 – small; d = .50 - medium; d = 0.80 – large; d = 1.20 – very large; d = 2.00 – huge. p-value is significant at < 0.05 (marked with *). TAU-U: TAU-U= < 0.20 – weak, TAU-U = <0.60 – moderate, TAU-U = <.80 – strong, TAU-U = <1.00 – very strong.

**Table 19-20.** Overview individual total scores and RCI’s for positive and negative affection scale (PANAS)

| **Pp** | **T0** | **T1** | **T2** | **RCI**  **T0-T1** | **RCI**  **T1-T2** | **RCI**  **T0-T2** |
| --- | --- | --- | --- | --- | --- | --- |
| **1** | 8 | 21 | 21 | 5.97 | 0 | 5.97 |
| **2** | 24 |  |  |  |  |  |
| **3** | 27 |  |  |  |  |  |
| **4** | 13 | 15 |  | 9.03 |  |  |
| **5** | 10 | 17 | 4 | 3.22 | -4.71 | -0.60 |
| **6** | 28 |  |  |  |  |  |
| **7** | 27 |  |  |  |  |  |
| **8** | 25 | 30 | 16 | 2.29 | -6.43 | -4.14 |
| **9** | 19 | 18 | 3 | -0.46 | -5.43 | -7.35 |
| **10** | 34 | 34 | 33 | 0 | -0.46 | -0.46 |
| **11** | 23 |  |  |  |  |  |
| **12** | 18 | 22 | 26 | 1.84 | 1.84 | 3.68 |
| **13** | 9 |  |  |  |  |  |
| **14** | 21 | 17 | 19 | -1.84 | 0.92 | -0.92 |
| **15** | 12 | 21 | 24 | 4.14 | 1.38 | 5.52 |
| **2** | 29 | 24 | 30 | -2.30 | 2.76 | 0.46 |
| **7** | 8 | 20 | 10 | 5.51 | -4.60 | 0.92 |
| **18** | 9 | 18 | 9 | 4.14 | -4.14 | 0 |
| **19** | 18 | 25 | 32 | 3.22 | 3.22 | 6.43 |
| **20** | 23 | 26 | 30 | 1.38 | 1.84 | 3.22 |
| **21** | 23 |  |  |  |  |  |
| **22** | 18 | 24 | 27 | 2.76 | 1.38 | 4.14 |

| **Pp** | **T0** | **T1** | **T2** | **RCI**  **T0-T1** | **RCI**  **T1-T2** | **RCI**  **T0-T2** |
| --- | --- | --- | --- | --- | --- | --- |
| **1** | 22 | 14 | 20 | -3.33 | 2.50 | -0.83 |
| **2** | 8 |  |  |  |  |  |
| **3** | 9 |  |  |  |  |  |
| **4** | 1 | 1 |  | 0 |  |  |
| **5** | 6 | 2 | 3 | -1.66 | 0.42 | 0.42 |
| **6** | 0 |  |  |  |  |  |
| **7** | 3 |  |  |  |  |  |
| **8** | 14 | 16 | 7 | 0.83 | 3.75 | -2.92 |
| **9** | 5 | 2 | 6 | -1.25 | 1.67 | 0.42 |
| **10** | 20 | 17 | 19 | -1.25 | 0.83 | -0.42 |
| **11** | 1 |  |  |  |  |  |
| **12** | 17 | 17 | 16 | 0 | -0.42 | -0.42 |
| **13** | 9 |  |  |  |  |  |
| **14** | 11 | 13 | 15 | 0.83 | 0.83 | 1.67 |
| **15** | 5 | 4 | 3 | -0.42 | -0.42 | -0.83 |
| **16** | 7 | 8 | 8 | 0.42 | 0 | 0.42 |
| **17** | 28 | 16 | 26 | -5.00 | 4.17 | -0.83 |
| **18** | 23 | 36 | 22 | 5.42 | -5.83 | -0.42 |
| **19** | 0 | 5 | 14 | 2.08 | 3.75 | 5.83 |
| **20** | 19 | 25 | 22 | 2.50 | -1.25 | 1.25 |
| **21** | 13 |  |  |  |  |  |
| **22** | 13 | 8 | 18 | -2.08 | 4.17 | 2.08 |

**Table 19.** Positive Affect Subscale **Table 20.** Negative Affect Subscale

***Note.*** RCI = significant at level < -1.96 or > 1.96.

**Table 21-22.** Overview scores individual total scores and RCI’s for self-reflection and insight-scale (SRIS-Y)

**Table 21.** Self-reflection subscale **Table 22.** Insight subscale

| **Pp** | **T0** | **T1** | **T2** | **RCI**  **T0-T1** | **RCI**  **T1-T2** | **RCI**  **T0-T2** |
| --- | --- | --- | --- | --- | --- | --- |
| **1** | 38 | 44 | 41 | 2.65 | -1.33 | 1.33 |
| **2** | 45 |  |  |  |  |  |
| **3** | 38 |  |  |  |  |  |
| **4** | 40 | 49 |  | 3.98 |  |  |
| **5** | 37 | 26 | 35 | -4.87 | 3.98 | -0.88 |
| **6** | 36 |  |  |  |  |  |
| **7** | 44 |  |  |  |  |  |
| **8** | 47 | 41 | 34 | -2.65 | -3.10 | -5.42 |
| **9** | 37 | 30 | 35 | -3.10 | 2.22 | -0.88 |
| **10** | 39 | 41 | 52 | 0.88 | 4.87 | 5.75 |
| **11** | 47 |  |  |  |  |  |
| **12** | 45 | 48 | 39 | 1.33 | -3.98 | -2.65 |
| **13** | 34 |  |  |  |  |  |
| **14** | 39 | 42 | 43 | 1.33 | 0.44 | 1.77 |
| **15** | 32 | 37 | 45 | 2.21 | 3.54 | 5.76 |
| **16** | 44 | 40 | 53 | -1.77 | 5.75 | 3.98 |
| **17** | 33 | 34 | 42 | 0.44 | 3.54 | 3.98 |
| **18** | 38 | 37 | 37 | -0.44 | 0 | -0.44 |
| **19** | 54 | 42 | 62 | -5.30 | 8.85 | 3.54 |
| **20** | 47 | 45 | 48 | -0.88 | 1.33 | 0.44 |
| **21** | 55 |  |  |  |  |  |
| **22** | 38 | 46 | 44 | 3.54 | -0.88 | 2.65 |

| **Pp** | **T0** | **T1** | **T2** | **RCI**  **T0-T1** | **RCI**  **T1-T2** | **RCI**  **T0-T2** |
| --- | --- | --- | --- | --- | --- | --- |
| **1** | 15 | 18 | 15 | 1.22 | -1.22 | 0 |
| **2** | 20 |  |  |  |  |  |
| **3** | 27 |  |  |  |  |  |
| **4** | 31 | 32 |  | 0.41 |  |  |
| **5** | 27 | 25 | 29 | -0.82 | 1.63 | 0.82 |
| **6** | 25 |  |  |  |  |  |
| **7** | 29 |  |  |  |  |  |
| **8** | 17 | 17 | 24 | 0 | 2.86 | 2.86 |
| **9** | 30 | 32 | 29 | 0.82 | -1.22 | -0.41 |
| **10** | 22 | 17 | 25 | -2.04 | 1.22 | 1.22 |
| **11** | 24 |  |  |  |  |  |
| **12** | 17 | 21 | 18 | 1.63 | 1.22 | 0.41 |
| **13** | 27 |  |  |  |  |  |
| **14** | 27 | 26 | 25 | -0.41 | -0.41 | 0.82 |
| **15** | 31 | 26 | 19 | -2.04 | -2.86 | -4.90 |
| **16** | 19 | 18 | 21 | -0.41 | 1.22 | 0.82 |
| **17** | 18 | 17 | 21 | -0.41 | 1.63 | 1.22 |
| **18** | 15 | 14 | 15 | -0.41 | 0.41 | 0 |
| **19** | 35 | 26 | 28 | -3.67 | 0.82 | -2.86 |
| **20** | 19 | 21 | 22 | 0.82 | 0.41 | 1.22 |
| **21** | 30 |  |  |  |  |  |
| **22** | 21 | 22 | 21 | 0.41 | -0.41 | 0 |

***Note.*** RCI = significant at level < -1.96 or > 1.96.

**Table 23.** Overview scores individual total scores and RCI’s for emotional awareness (MAIA)

| **Pp** | **T0** | **T1** | **T2** | **RCI**  **T0-T1** | **RCI**  **T1-T2** | **RCI**  **T0-T2** |
| --- | --- | --- | --- | --- | --- | --- |
| **1** | 4 | 3,6 | 4,4 | -0.43 | 0.87 | 0.43 |
| **2** | 4,2 |  |  |  |  |  |
| **3** | 4 |  |  |  |  |  |
| **4** | 1,4 |  |  |  |  |  |
| **5** | 2,8 | 0,2 | 1,6 | -2.83 | 1.52 | -1.30 |
| **6** | 3,8 |  |  |  |  |  |
| **7** | 1,6 |  |  |  |  |  |
| **8** | 4,8 | 4,6 | 4,2 | -0.22 | -0.43 | -0.65 |
| **9** | 3 | 2,2 | 4,4 | -0.87 | 2.39 | 1.52 |
| **10** | 4,4 | 5 | 5 | 0.65 | 0 | 0.65 |
| **11** | 2,2 |  |  |  |  |  |
| **12** | 3 | 2,2 | 3 | -0.86 | 0.87 | 0 |
| **13** | 3,4 |  |  |  |  |  |
| **14** | 2,2 | 2,8 | 2 | 0.65 | -0.87 | -0.22 |
| **15** | 4 | 5 | 5 | 1.08 | 0 | 1.09 |
| **16** | 3,2 | 1,8 | 3,2 | 1.52 | 1.52 | 0 |
| **17** | 2,6 | 3,4 | 3,2 | 0.87 | -0.22 | 0.65 |
| **18** | 1 | 1,8 | 2,6 | 0.87 | 0.87 | 1.74 |
| **19** | 3,6 | 4 | 3,8 | 0.43 | -0.22 | 0.22 |
| **20** | 4,2 | 4,4 | 4,8 | 0.22 | 0.43 | 0.65 |
| **21** | 4,4 |  |  |  |  |  |
| **22** | 4 | 3,6 | 3,4 | -0.43 | -0.22 | -0.65 |

***Note.*** RCI = significant at level < -1.96 or > 1.96.

**Table 24.** Overview scores individual total scores and RCI’s for treatment motivation (ATMQ)

| **Pp** | **T0** | **T1** | **T2** | **RCI**  **T0-T1** | **RCI**  **T1-T2** | **RCI**  **T0-T2** |
| --- | --- | --- | --- | --- | --- | --- |
| **1** | 2,45 | 2,82 | 2,73 | 0.54 | -0.13 | 0.41 |
| **2** | 2,2 |  |  |  |  |  |
| **3** | 2,09 |  |  |  |  |  |
| **4** | 1,82 | 2,00 |  | 0.26 |  |  |
| **5** | 2,09 | 2,18 | 1,64 | 0.13 | -0.79 | -0.66 |
| **6** | 1,7 |  |  |  |  |  |
| **7** | 2,4 |  |  |  |  |  |
| **8** | 2,5 | 2,55 | 2,91 | 0.07 | 0.53 | 0.60 |
| **9** | 2,3 | 2,27 | 2,18 | -0.04 | -0.13 | -0.18 |
| **10** | 2,5 | 2,36 | 2,27 | -0.20 | -0.13 | -0.34 |
| **11** | 2,2 |  |  |  |  |  |
| **12** | 2,3 | 2,18 | 2,64 | -0.18 | 0.68 | 0.50 |
| **13** | 2,5 |  |  |  |  |  |
| **14** | 2,3 | 2,73 | 2,55 | 0.63 | -0.26 | 0.38 |
| **15** | 2,4 | 2,82 | 2,73 | 0.62 | -0.13 | 0.48 |
| **16** | 2,5 | 2,36 | 2,55 | -0.14 | 0.28 | 0.07 |
| **17** | 2,4 | 2,00 | 1,82 | -0.59 | -0.26 | -0.85 |
| **18** | 2,4 | 2,36 | 2,00 | -0.06 | -0.53 | -0.59 |
| **19** | 3 | 3,00 | 3,00 | 0 | 0 | 0 |
| **20** | 2,4 | 2,73 | 2,91 | 0.49 | 0.26 | 0.75 |
| **21** | 1,8 |  |  |  |  |  |
| **22** | 2,6 | 2,18 | 2,64 | -0.62 | 0.68 | 0.06 |

***Note.*** RCI = significant at level < -1.96 or > 1.96.

**Table 25.** Overview scores individual total scores and RCI’s treatment alliance (WAV-12)

| **Pp** | **T0** | **T1** | **T2** | **RCI**  **T0-T1** | **RCI**  **T1-T2** | **RCI**  **T0-T2** |
| --- | --- | --- | --- | --- | --- | --- |
| **1** | 57 | 57 | 58 | 0 | 0.33 | 0.33 |
| **2** | 56 |  |  |  |  |  |
| **3** | 44 |  |  |  |  |  |
| **4** | 28 | 31 |  | 0.98 |  |  |
| **5** | 33 | 18 | 24 | -4.92 | 1.96 | -2.95 |
| **6** | 34 |  |  |  |  |  |
| **7** | 56 |  |  |  |  |  |
| **8** | 57 | 58 | 58 | 0.33 | 0 | 0.33 |
| **9** | 49 | 55 | 57 | 1.97 | 0.66 | 2.62 |
| **10** | 49 | 58 | 59 | 2.95 | 0.33 | 3.28 |
| **11** | 43 |  |  |  |  |  |
| **12** | 44 | 42 | 41 | -0.66 | -0.33 | -0.98 |
| **13** | 54 |  |  |  |  |  |
| **14** | 45 | 49 | 39 | 1.31 | -3.28 | -1.97 |
| **15** | 60 | 59 | 54 | -0.33 | -1.64 | -1.97 |
| **16** | 52 | 37 | 47 | -4.92 | 3.28 | -1.64 |
| **17** | 56 | 36 | 41 | -6.56 | 1.64 | -4.92 |
| **18** | 42 | 45 | 36 | 0.98 | -2.95 | -1.97 |
| **19** | 58 | 42 | 57 | -5.25 | 3.93 | -0.33 |
| **20** | 51 | 55 | 51 | 1.31 | -1.31 | 0 |
| **21** | 40 |  |  |  |  |  |
| **22** | 39 | 45 | 44 | 1.97 | -0.33 | 1.64 |

***Note****.* RCI = significant at level < -1.96 or > 1.96.
