# Supplementary material for: ‘I gotta Feeling’: Exploring the effects of a smartphone app (Feelee) to enhance adolescents’ emotion regulation in forensic outpatient settings: A multiple single-case experimental design
Source: PLoS One. 2026 Feb 6;21(2):e0332111. doi: 10.1371/journal.pone.0332111 (PMC12880710; doi:10.1371/journal.pone.0332111)
Supplement: S3 File — (DOCX) [file pone.0332111.s003.docx]

**Appendix 3 -** Results of Mila

*The name presented in this case description has been pseudonymized.*
 **1. Profile**

Mila was a 19-year-old girl receiving personal support and trauma-focused treatment related to her safety and her vulnerability to delinquent behavior. At inclusion, she had been in treatment for three months, following a longer history of care. During the intervention phase, her clinician reported consistently discussing both the active emotion data (emoji-based check-ins) and the passive behavioral data from the Feelee app on a weekly basis. The clinician also encouraged her to use the app to gain more insight into her emotions throughout the week.

**2. Study conditions**

The baseline phase lasted 15 days, during which Mila completed 8 out of 15 daily questionnaires (53%). Following this, the intervention phase began, meaning Mila installed and started using the Feelee app alongside treatment as usual. Mila submitted an emoji on 16 out of 28 days (57%), and except for two of those days, she reported feeling ‘good’. She mostly used the Feelee app while on the way or during therapy sessions. In most entries, she indicated that she was not alone, but accompanied by her clinician, roommates, or classmates. According to the clinician's responses on the treatment integrity questionnaire, they succeeded in discussing both the active emotion (emoji) and passive behavioral data from the Feelee app weekly throughout the 28-day (4-week) intervention phase. During this period, Mila completed 17 out of 28 daily questionnaires (61%). At the start of the follow-up phase, Mila uninstalled the Feelee app. The follow-up lasted 14 days, during which she completed 8 out of 14 daily questionnaires (57%). Finally, Mila completed all measurement points: pre-test (T0), post-test (T1), follow-up (T2), and 3-month follow-up (T3).

**3. Primary outcome**

*a. Recognition items*

For the first item, emotion recognition (clarity), an increase was expected. Visual inspection of Mila’s individual scores showed only limited changes across phases (Figure 1). The randomization test nevertheless indicated a significant effect between baseline and intervention (p = 0.003) and between intervention and follow-up (p = 0.004). The comparison between baseline and follow-up was also significant (p = 0.007). In line with this, the TAU-U analyses showed a notable non-overlap between baseline and follow-up (p = 0.018). For suppression, scores decreased from baseline to post-intervention, although this change was not statistically significant based on visual analysis (Figure 2). The TAU-U scores, however, supported this downward trend by showing a significant non-overlap between baseline and intervention (p = 0.009).

*b. Reflection items*

Regarding the reflection-related items, an increase in scores on both items was anticipated. Instead, Mila’s rumination scores decreased (Figure 4). The randomization test did not show a significant decrease between baseline and intervention, although reversed testing indicated significant effects between baseline and intervention (p = 0.004) and between intervention and follow-up (p = 0.006). TAU-U analyses also showed significant non-overlap between baseline and intervention (p = 0.001). Reappraisal followed a similar pattern. Reversed testing showed significant effects from baseline to intervention (p = 0.002) and from intervention to follow-up (p = 0.005), while the TAU-U results again indicated significant non-overlap only between baseline and intervention (p = 0.001).

*c. Managing items*

For managing emotions, both impulsivity control and distraction were expected to increase. Visual analysis of impulsivity showed a slight increase during baseline, followed by stable scores during intervention and follow-up (Figure 5). The randomization test did not indicate significant changes across phases. However, the slight increase during baseline was reflected in the TAU-U analyses, which showed significant non-overlap between baseline and intervention (p = 0.02). For distraction, visual analysis revealed substantial day-to-day variability and a clear decline between baseline and intervention (Figure 6). The randomization test did not show significant increases across phases, yet reversed testing identified small but significant decreases between baseline and intervention (p = 0.04) and between intervention and follow-up (p = 0.03). Additionally, significant non-overlap was found between baseline and intervention (p = 0.022) and between baseline and follow-up (p = 0.026).

**Figure 1. Emotional recognition (clarity)**Item 1. In the past 24 hours, I had no idea how I was feeling - almost never (0) → almost always (5).

**
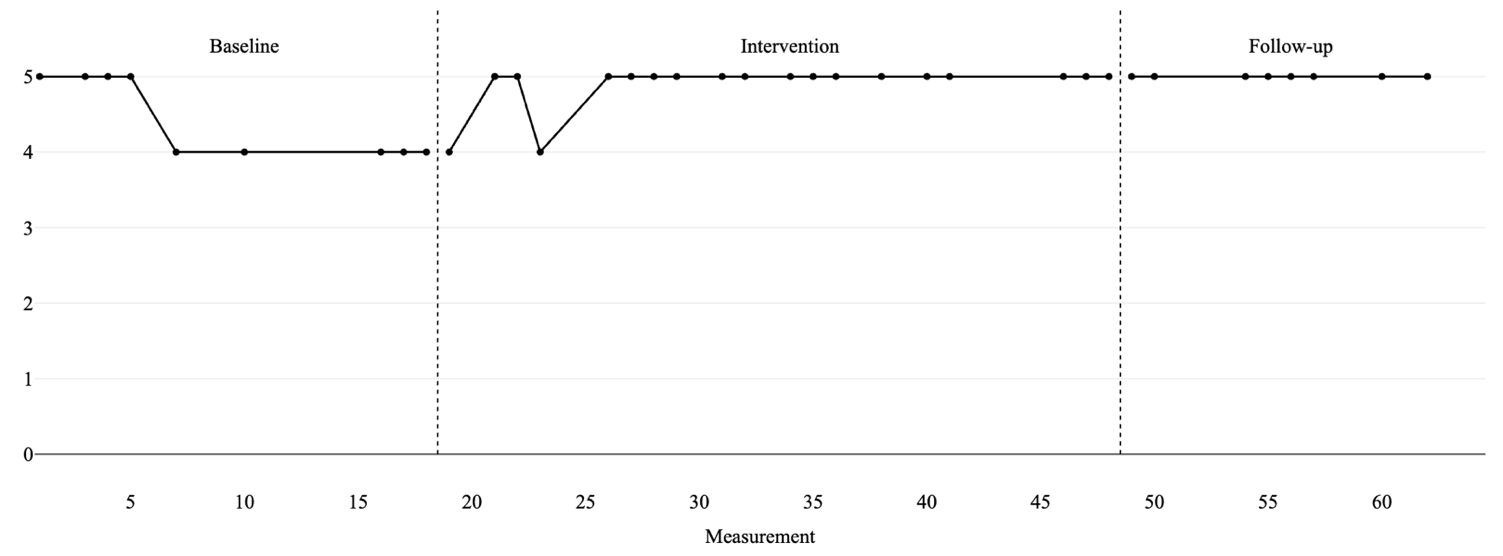
***Note. Item was reverse-coded, expected direction: increase*

**Figure 2. Emotional suppression**Item 2. In response to my emotions, I pretended I wasn’t upset – not at all (0) → very much (10)

**
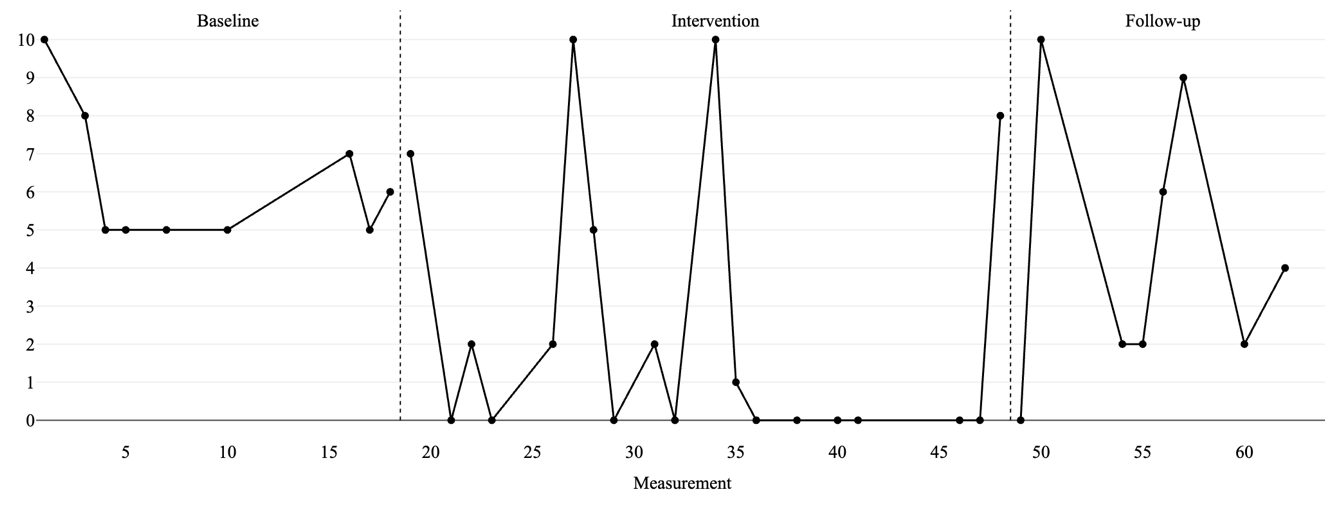
**


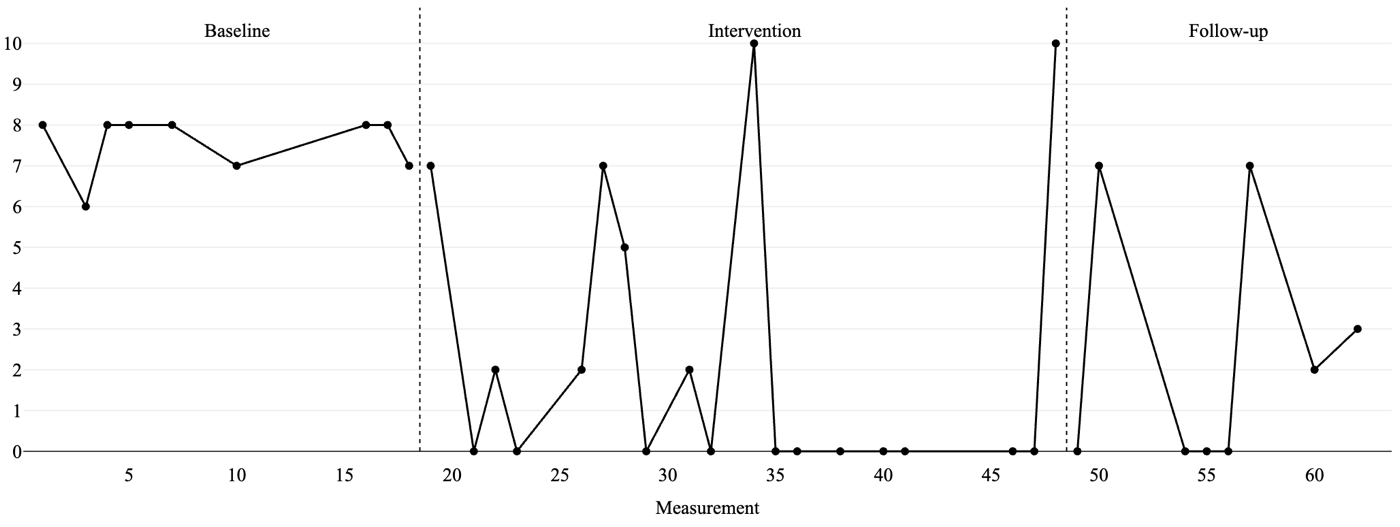
**Figure 3. Reflection, comprehense (rumination)**Item 3. In response to my emotions, I thought of other ways to interpret the situation – not at all (0) → very much (10)

**Figure 4. Reflection, comprehense (reappraisal)**Item 4. In response to my emotions, I thought of other ways to interpret the situation – not at all (0) → very much (10)


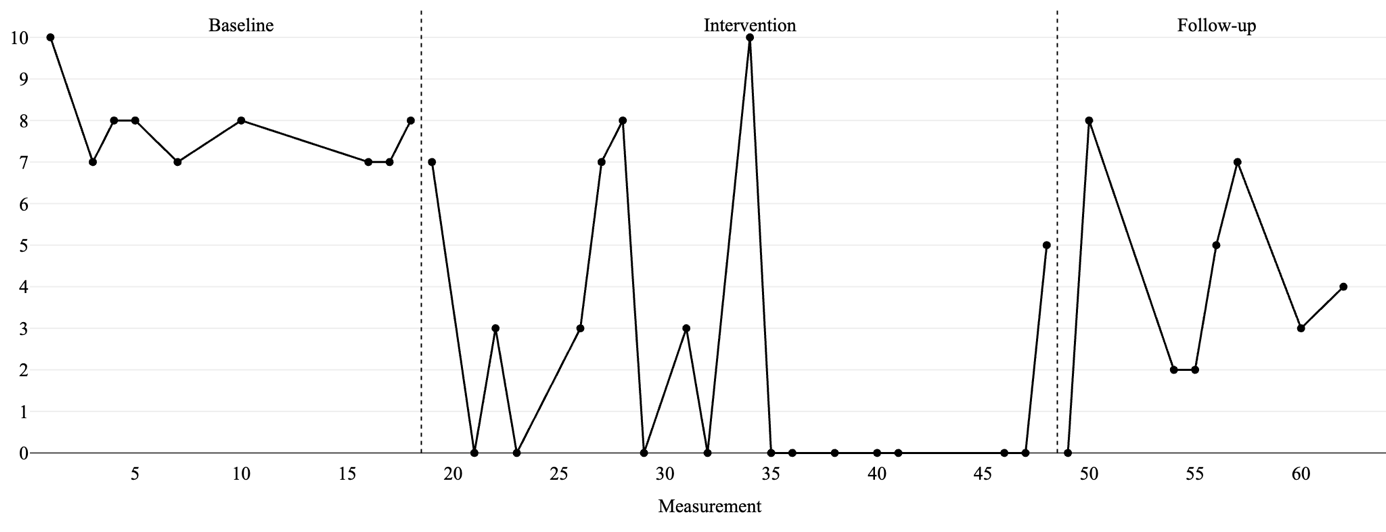


**Figure 5. Managing (impulse)**Item 5. In the past 24 hours, when I'm upset, I had difficulty controlling my behaviors - almost never (0) → almost always (5)


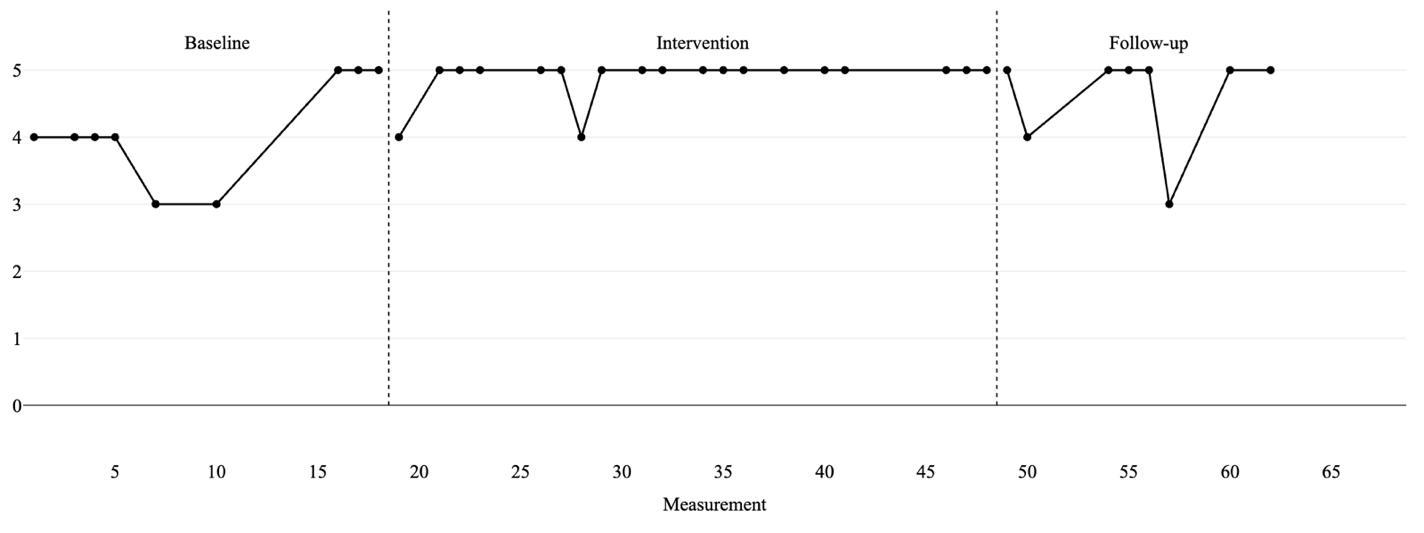


*Note. Item was reverse-coded, expected direction: increase* **Figure 6. Managing (distraction)**Item 6. In response to my emotions, I engaged in activities to distract myself – not at all (0) → very much (10)


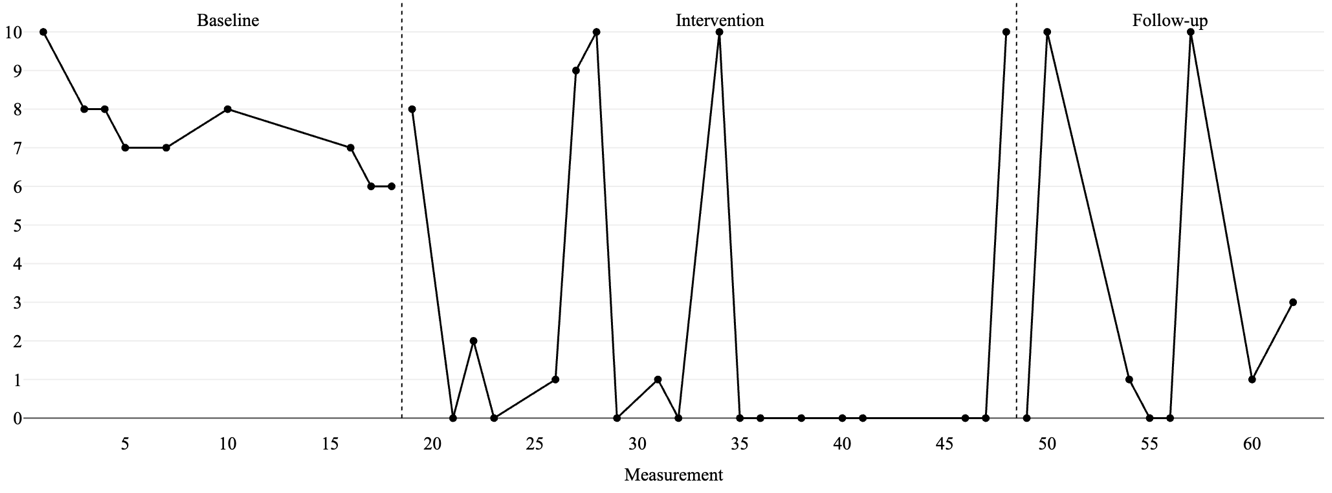


**4. Secondary outcomes**

The secondary outcomes related to emotional reflection show a pattern similar to the primary reflection items. Across the study, RCI-scores indicate a reliable decrease in reflection (SRIS-Y). However, for insight (SRIS-Y), scores increased between T1–T2 and T0–T2. RCI’s scores indicated that these scores were reliable. Regarding treatment factors, no significant changes were observed in either motivation (ATMQ) or therapeutic alliance (WAV-12). On both scales, Mila reported consistently high scores with no notable changes throughout the study.

**Tabel 1.** Results emotional factors

|  | **T0** | **T1** | **T2** | **RCI T0-T1** | **RCI T1-T2** | **RCI T0-T2** |
| --- | --- | --- | --- | --- | --- | --- |
| Positive affect (PANAS) | 25 | 30 | 16 | 2.29 | -6.43 | -4.14 |
| Negative affect (PANAS) | 14 | 16 | 7 | 0.83 | 3.75 | -2.92 |
| Self-reflection (SRIS-Y) | 47 | 41 | 34 | -2.65 | -3.10 | -5.42 |
| Insight (SRIS-Y) | 17 | 17 | 24 | 0 | 2.86 | 2.86 |
| Emotional awareness (MAIA) | 4,8 | 4,6 | 4,2 | -0.22 | -0.43 | -0.65 |

Note. RCI = significant at level < -1.96 or > 1.96.

**Tabel 2.** Results treatment factors

|  | **T0** | **T1** | **T2** | **RCI T0-T1** | **RCI T1-T2** | **RCI T0-T2** |
| --- | --- | --- | --- | --- | --- | --- |
| Treatment motivation (ATMQ) | 2,5 | 2,55 | 2,91 | 0.07 | 0.60 | 0.53 |
| Treatment alliance (WAV-12) | 57 | 58 | 58 | 0.33 | 0.33 | 0 |

Note. RCI = significant at level < -1.96 or > 1.96.

**5. Qualitative results**

In the interview, Mila said the Feelee app did not help her understand her emotions better. However, it provided her more overview and structure. The involved clinicians noted that Mila could already identify her emotions well at the start of the intervention. Still, she often forgot how she had felt or reinterpreted situations later. She reacted emotionally in the moment but could not recall the reason a week later. As the clinician said:

*“(...) I think she also said about herself that, sometimes afterwards it turns out to be very different from how it felt in the moment. That in the moment, it can feel really overwhelming”* (C3).

Mila said Feelee helped her remember how she had felt the day before and reflect on it. For both positive and negative emotions, she later said it “wasn’t that bad” or that she had reflected more afterward:

*“Because when I, like, when the next day I’m feeling a different emotion, I still think back to yesterday—which I never used to do. Then I’m like, (...) ‘Wow, how did I think that at the time?’ And later I think again, ‘How was I really thinking then?”*
