# Supplementary material for: ‘I gotta Feeling’: Exploring the effects of a smartphone app (Feelee) to enhance adolescents’ emotion regulation in forensic outpatient settings: A multiple single-case experimental design
Source: PLoS One. 2026 Feb 6;21(2):e0332111. doi: 10.1371/journal.pone.0332111 (PMC12880710; doi:10.1371/journal.pone.0332111)
Supplement: S4 File — (DOCX) [file pone.0332111.s004.docx]

**Appendix 4 -** Results of Vidar

*The name presented in this case description has been pseudonymized.*
 **1. Profile**

Vidar is a 16-year-old boy under juvenile probation following a court conviction and a period of detention in a secure facility. At the start of the study, he had been receiving individual treatment for 14 months, focusing on offense analysis and systemic therapy. His participation aimed to strengthen his understanding of emotions. Regarding treatment integrity during the intervention phase, the clinician reported discussing the Feelee data twice, once during an in-person session and once by phone.

**2. Study conditions**

During the 14-day baseline, Vidar completed 8 out of 14 daily questionnaires in the Feelee app (57%). The intervention phase lasted 31 days, during which he selected an emoji in the app 16 times (52%). Except for two entries, Vidar consistently reported feeling ‘good’, most often attributing this to ‘having a good night of sleep’. He most frequently completed the Feelee check-in while ‘relaxing’ and being ‘alone’. Furthermore, he completed 15 of 31 daily questionnaires (48%). Follow-up lasted 21 days, with 8 of 21 questionnaires completed (38%). Feelee data were discussed once in session and twice via phone, though the latter focused only on app use. Vidar completed pre-test (T0), post-test (T1), follow-up (T2), but declined to participate in the 3-months follow-up (T3) measurement.

**3. Primary outcome**

*a. Recognition items*

Regarding the first step of emotion regulation, recognition (clarity) an increase was expected across baseline, intervention and follow-up. Vidar’s scores, however, fluctuated considerably throughout the study period (Figure 1). The randomization test did not reveal significant changes between phases. Even so, the TAU-U analysis pointed to a meaningful non-overlap between baseline and follow-up (p = 0.026), suggesting some gradual improvement over time. For suppression, a reduction was anticipated. Vidar showed the opposite pattern: suppression increased during the intervention compared to baseline (Figure 2). This upward shift was supported by the randomization test, with significant differences emerging between baseline and intervention (p = 0.001) and between intervention and follow-up (p = 0.003). TAU-U scores similarly showed a significant non-overlap between baseline and intervention (p = 0.015), while follow-up scores stabilized.

*b. Reflection items*

For the reflection items, increases in both rumination and reappraisal were expected. Rumination scores, however, showed notable variability without a clear upward or downward trajectory (Figure 3). Neither the randomization test nor the TAU-U analysis detected meaningful changes across phases. Reappraisal followed a somewhat different course. Visual inspection revealed high scores during baseline, followed by a sharp drop at the next measurement point (Figure 4), coinciding with reduced app engagement that week. While the randomization test did not show significant shifts across phases, reversed testing indicated significant decreases between intervention and follow-up (p = 0.002) and between baseline and follow-up (p = 0.002). No additional non-overlap effects emerged from the TAU-U analyses.

*c. Managing items*

Regarding the managing items, increases in impulse control and distraction were anticipated. Vidar began with consistently high impulsivity scores during baseline and the early intervention phase, after which more variability appeared in his responses (Figure 5). Randomization testing did not show significant differences across phases. The TAU-U analysis, however, indicated a significant non-overlap between intervention and follow-up (p = 0.004), suggesting some improvement toward the end of the study. Distraction scores were high during baseline and the early intervention weeks, after which they declined and stayed relatively low, with some variability, during follow-up (Figure 6). Although the randomization test did not reveal significant effects, TAU-U analyses identified meaningful non-overlap between baseline and intervention (p = 0.05) and between baseline and follow-up (p = 0.026), indicating a gradual decrease across the study period.


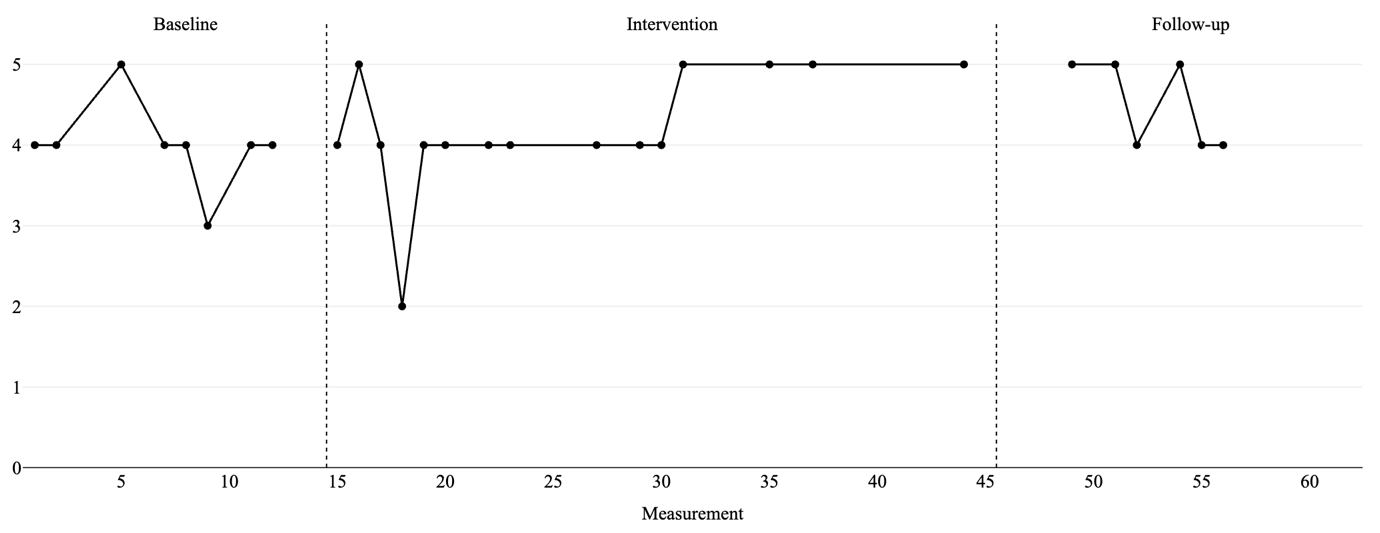
**Figure 1. Emotional recognition**Item 1. In the past 24 hours, I had no idea how I was feeling - almost never (0) → almost always (5)

*Note. Item was recoded, expected direction is an increase.*

**Figure 2. Emotional suppression**Item 2. In response to my emotions, I pretended I wasn’t upset – not at all (0) → very much (10)


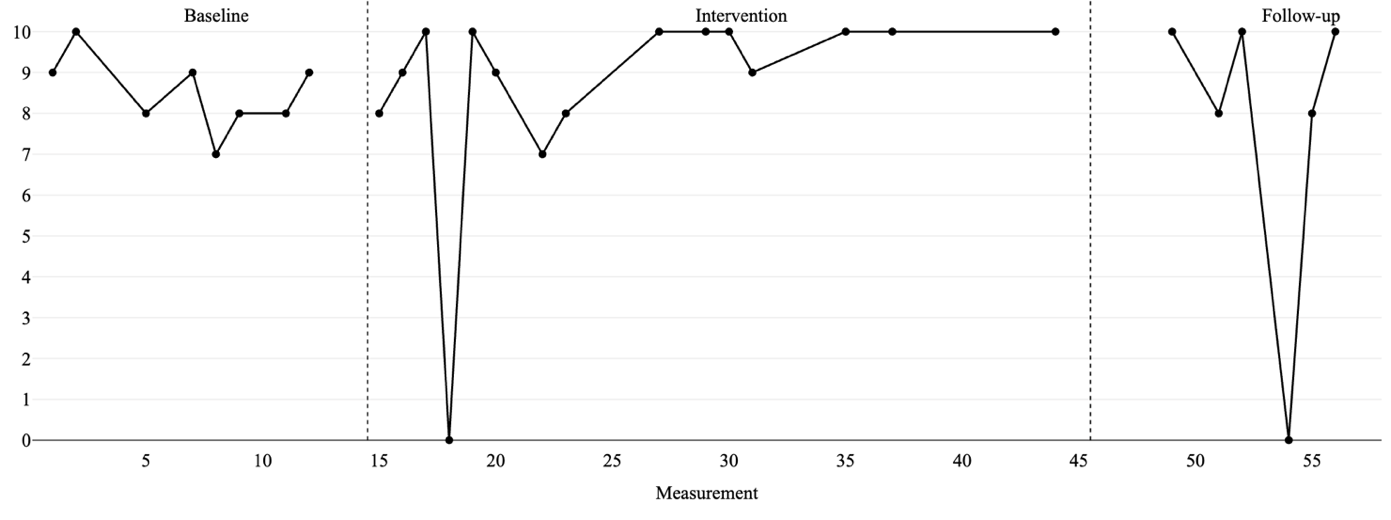


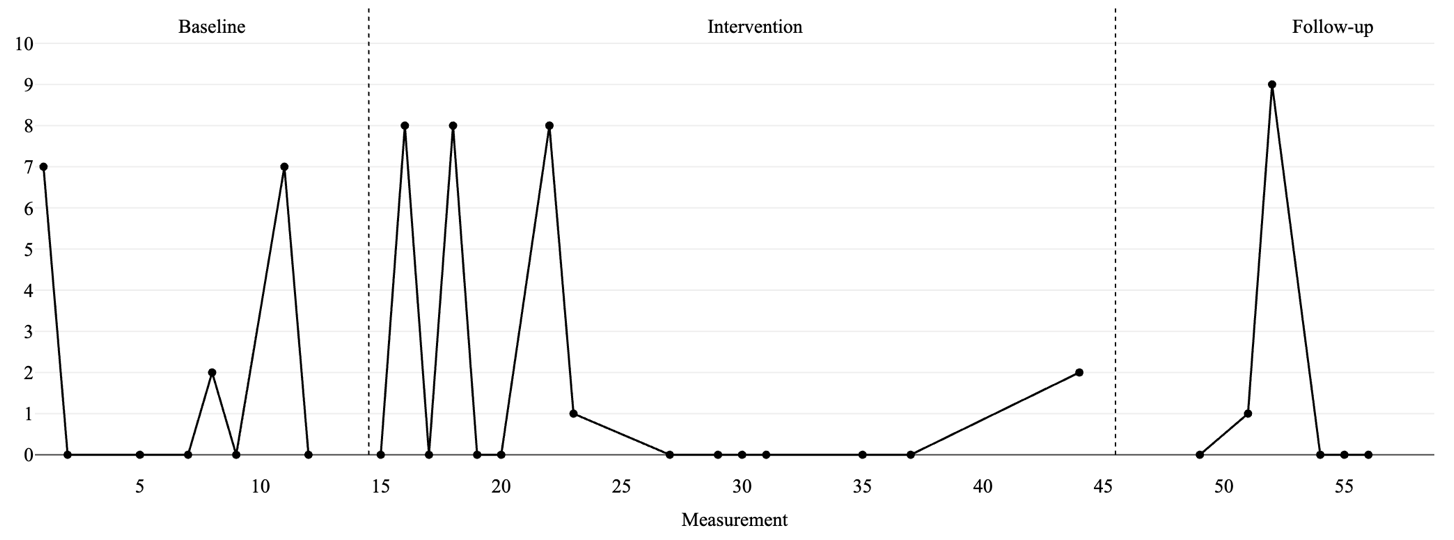
**Figure 3. Reflection, comprehense (rumination)**Item 3. In response to my emotions, I thought of other ways to interpret the situation – not at all (0) → very much (10)

**Figure 4. Reflection, comprehense (reappraisal)**Item 4. In response to my emotions, I thought of other ways to interpret the situation – not at all (0) → very much (10)


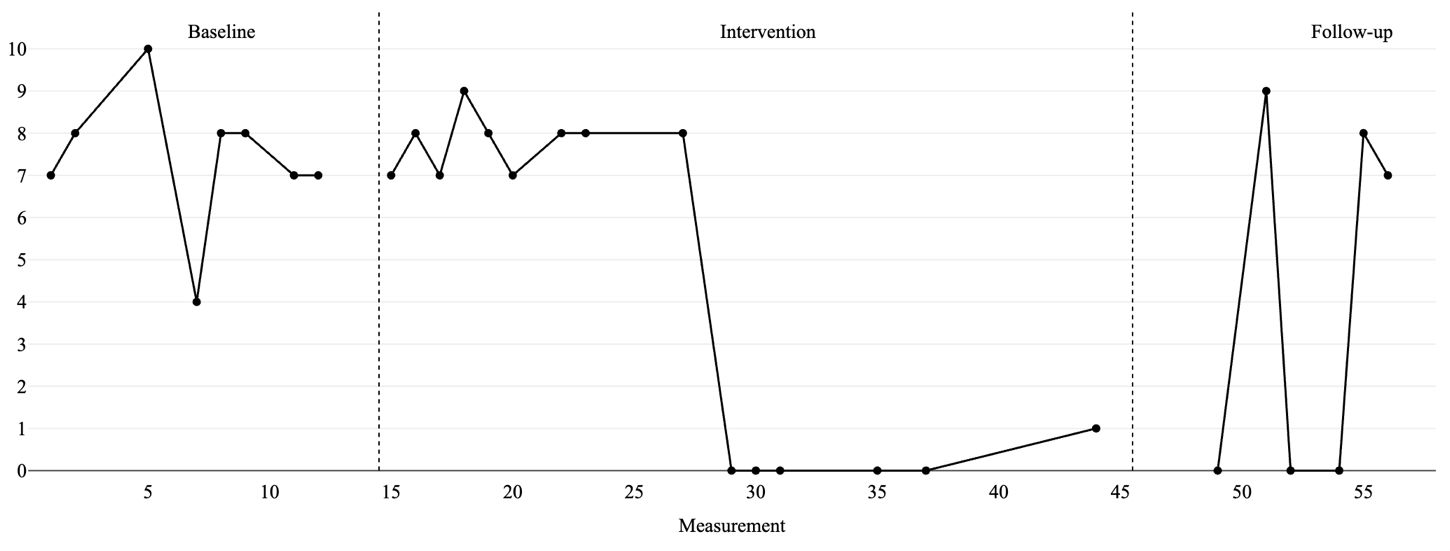


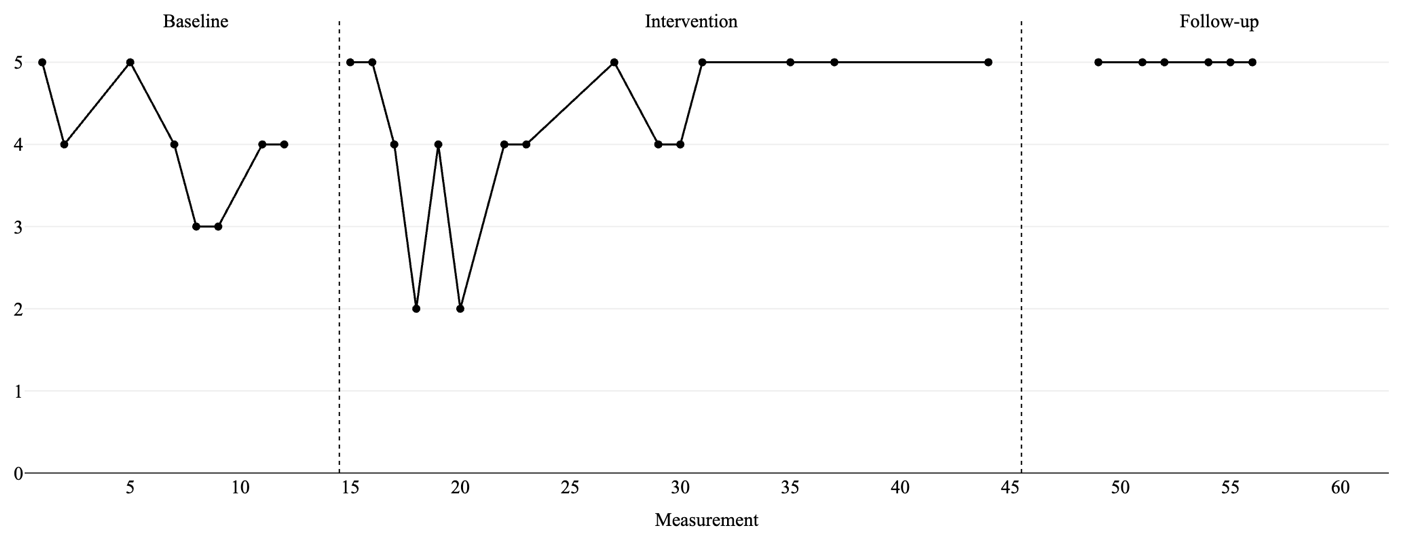
**Figure 6. Managing (impulse)**Item 6. In the past 24 hours, when I'm upset, I had difficulty controlling my behaviors- almost never (0) → almost always (5)

*Note. Item was recoded, expected direction is an increase.*


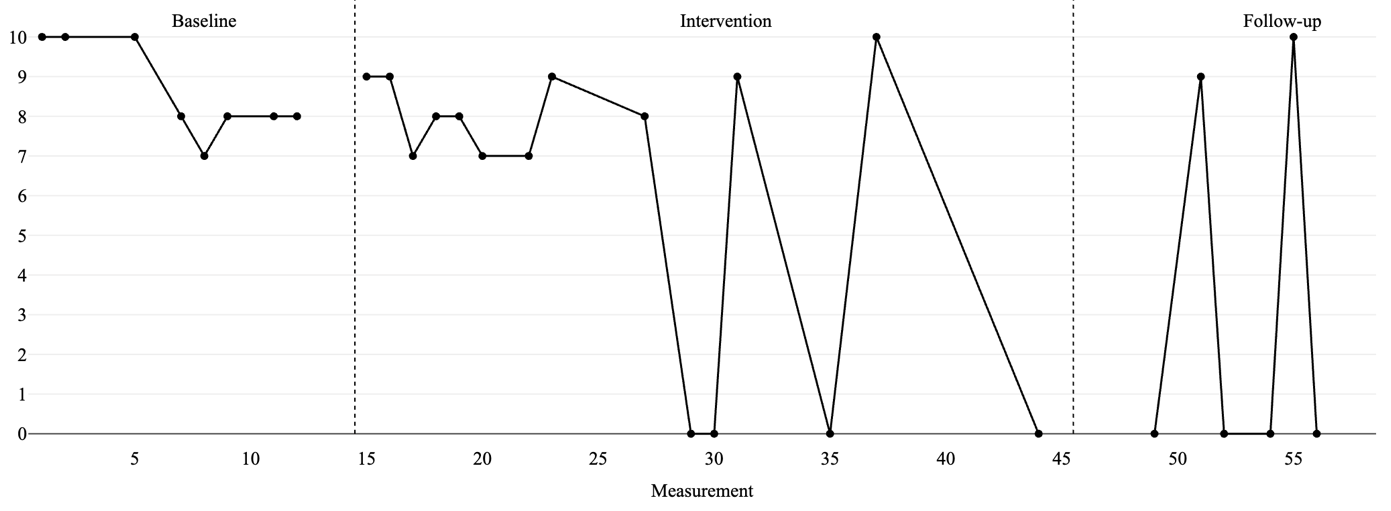
**Figure 5. Managing (distraction)**Item 5. In response to my emotions, I engaged in activities to distract myself – not at all (0) → very much (10)

**4. Secondary outcomes**

On secondary outcomes, Vidar showed a reliable decrease on reflection (SRIS-Y) between T0-T1, with no reliable change between T1-T2. However, RCI-scores between T0-T2 show a reliable increase on reflection. For insight (SRIS-Y), reliable increase was found between T0-T1, a small but not reliable decrease between T1-T2, which turns reliable between T0-T2 comparison. For emotional awareness (MAIA), small but not reliable scores were observed T1-T2, which turns reliable between T0-T2. This suggests a reliable increase of emotional awareness throughout study. For treatment factors, RCI scores showed no statistically significant change for treatment motivation (ATMQ). For treatment alliance (WAV-12), a reliable change was observed between al measurements, indicating a reliable increase in treatment alliance.

**Tabel 1.** Results emotional factors

|  | **T0** | **T1** | **T2** | **RCI T0-T1** | **RCI T1-T2** | **RCI T0-T2** |
| --- | --- | --- | --- | --- | --- | --- |
| Positive affect (PANAS) | 19 | 18 | 3 | -0.46 | -5.43 | -7.35 |
| Negative affect (PANAS) | 5 | 2 | 6 | -1.25 | 1.67 | 0.42 |
| Self-reflection (SRIS-Y) | 47 | 41 | 34 | -2.65 | -3.10 | -5.42 |
| Insight (SRIS-Y) | 30 | 32 | 29 | 0.82 | -1.22 | -0.41 |
| Emotional awareness (MAIA) | 3 | 2,2 | 4,4 | -0.87 | 2.39 | 1.52 |

Note. RCI = significant at level < -1.96 or > 1.96.

**Tabel 2.** Results treatment factors

|  | **T0** | **T1** | **T2** | **RCI T0-T1** | **RCI T1-T2** | **RCI T0-T2** |
| --- | --- | --- | --- | --- | --- | --- |
| Treatment motivation (ATMQ) | 2,3 | 2,18 | 2,64 | -0.18 | 0.50 | 0.68 |
| Treatment alliance (WAV-12) | 44 | 42 | 41 | -0.66 | -0.98 | -0.33 |

Note. RCI = significant at level < -1.96 or > 1.96.

**5. Qualitative results**

During the interview, Vidar stated that Feelee was helpful for him to get a better understanding of his emotions. As he explained:

*“It just made me think more, like, ‘Oh, yesterday I felt that way.’ Like, I had no energy, for example. I was angry or something.”*.

In treatment, Vidar indicates he rarely discussed the emotions, although he felt he could if he wanted to. Although, he marked that Feelee served in treatment as a memory aid to remind him of emotions and situations. As Vidar explained:

*"So, yeah… it’s like you go through the situation twice. You’ve already experienced it, but then when you fill in the app, you reflect on it again and that helps you remember it more clearly.”*

The involved clinician confirmed that Vidar frequently forgot to use the Feelee app and did not review the data he had entered by himself. Therefore, it was difficult to elaborate on his emotions during sessions.
