# Supplementary material for: ‘I gotta Feeling’: Exploring the effects of a smartphone app (Feelee) to enhance adolescents’ emotion regulation in forensic outpatient settings: A multiple single-case experimental design
Source: PLoS One. 2026 Feb 6;21(2):e0332111. doi: 10.1371/journal.pone.0332111 (PMC12880710; doi:10.1371/journal.pone.0332111)
Supplement: S5 File — (DOCX) [file pone.0332111.s005.docx]

**Appendix 5 -** Results of Adam

*The name presented in this case description has been pseudonymized.*

**1. Profile**

Adam was a 16-year-old boy who had been in treatment for four months at the time of inclusion. He had been convicted by a judge and had previously spent a period in a secure juvenile facility. At the start of the study, Adam was living at home, and his treatment focused on several areas, including family sessions and individual cognitive behavioral therapy. He also received outpatient support aimed at emotion regulation, mood, and behavioral activation. Given this focus, he was invited to participate in the Feelee study to support his therapeutic trajectory. The clinician involved reported that the Feelee data were discussed once during the four-week intervention phase.

**2. Study conditions**

For Adam, the baseline period lasted 16 days, during which he completed all 16 daily questionnaires. The intervention phase lasted 68 days. Despite a temporary interruption in treatment due to a relapse, Adam continued using the Feelee app. He submitted an emoji 49 times, which also included multiple submissions on the same day. In most cases, Adam reported feeling ‘okay’ (37 times) and indicated that he did not know why he felt that way. When submitting an emoji, he most often reported being alone. The involved clinician reported that Feelee data were discussed once during the intervention period. During intervention, Adam completed the daily questionnaire on 24 out of 68 days (35%). For the 14-day follow-up, he completed 10 out of 14 questionnaires (71%). Regarding secondary outcome measures, Adam completed the pre-test (T0), post-test (T1), and follow-up (T2), but declined participation in the three-month follow-up assessment (T3).

**3. Primary outcome**

*a. Recognition items*

On the first step of emotion regulation, an increase was expected on the first recognition (clarity) item. Visual inspection of Adam’s scores showed limited change across the study period (Figure 1), and the randomization test confirmed that no significant differences emerged between phases. TAU-U analyses likewise indicated no meaningful non-overlap effects. For suppression, Adam consistently reported high scores with little variation over time (Figure 2). The randomization test showed no significant differences between baseline and intervention or between baseline and follow-up. TAU-U analyses also revealed no significant non-overlap effects.

*b. Reflection items*

Regarding the reflection items, increases in rumination and reappraisal were anticipated. Rumination scores remained stable throughout the study (Figure 3), with no significant changes detected by the randomization test or TAU-U analyses. Reappraisal showed a somewhat different pattern. From measurement point 30 onward, a gradual decline was visible (Figure 4). The randomization test indicated a significant change between baseline and intervention (p = 0.03). However, this finding was not supported by the TAU-U analysis, which did not show significant non-overlap across phases.

*c. Managing items*

For the managing items, increases were expected on both impulsivity control and distraction. Adam reported consistently high impulsivity scores during baseline and the early intervention phase, followed by greater variability later in the study (Figure 5). Neither the randomization test nor the TAU-U analysis showed significant differences between phases. A similar pattern was observed for distraction (Figure 6). Scores remained relatively stable, and no significant changes or non-overlap effects were detected by either the randomization test or TAU-U analyses.

*
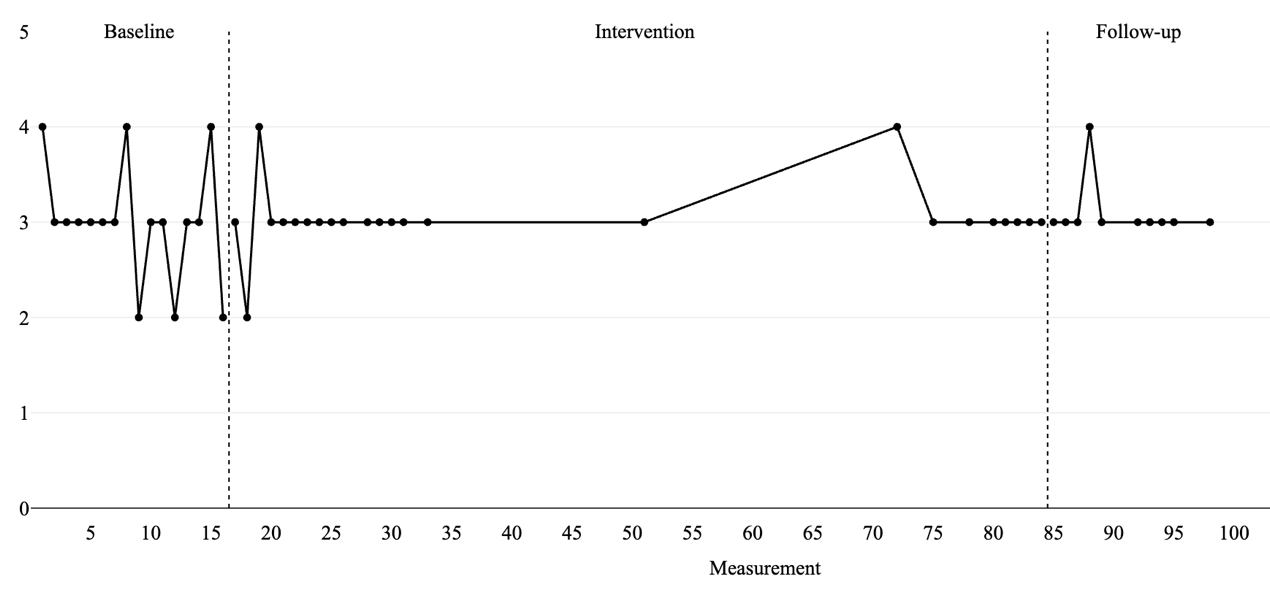
***Figure 1. Emotional recognition**Item 1. In the past 24 hours, I had no idea how I was feeling - almost never (0) → almost always (5)

*Note. Item was reverse-coded, expected direction: increase*

**
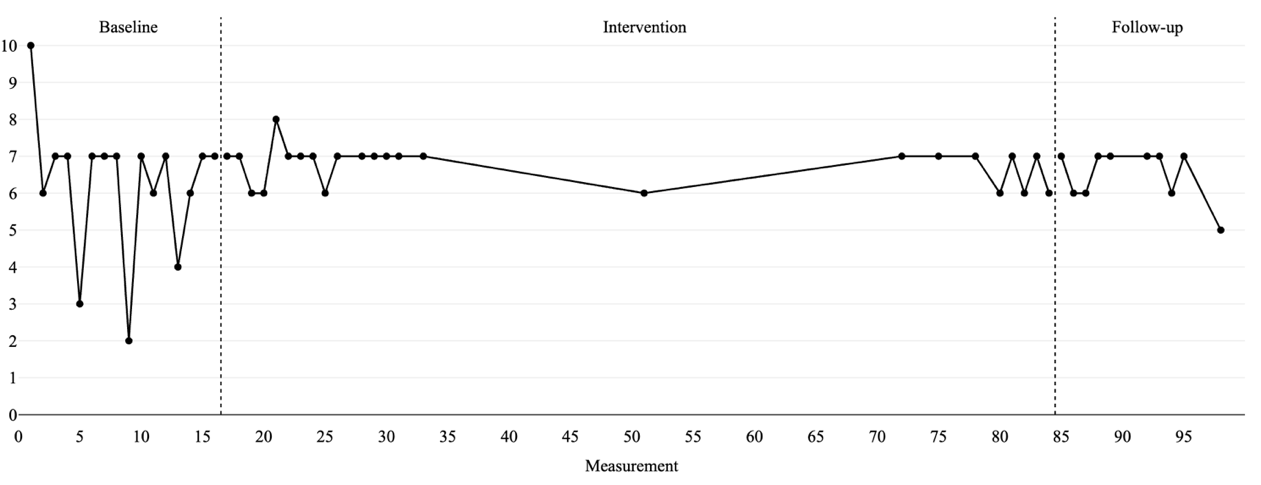
Figure 2. Emotional suppression**Item 2. In response to my emotions, I pretended I wasn’t upset – not at all (0) → very much (10)

**Figure 3. Reflection, comprehense (rumination)**Item 3. In response to my emotions, I thought of other ways to interpret the situation – not at all (0) → very much (10)

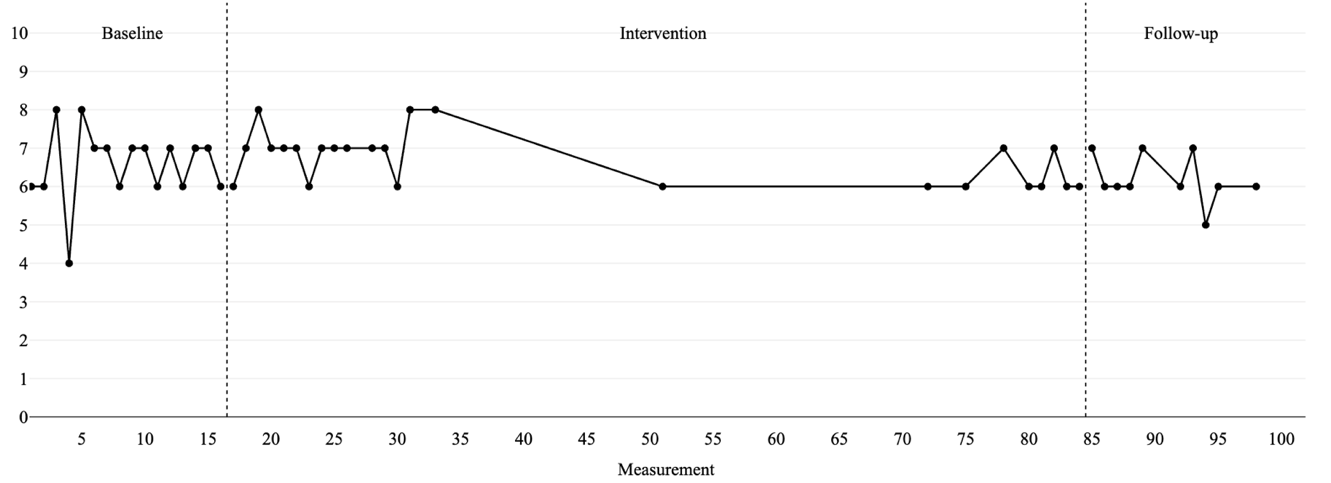


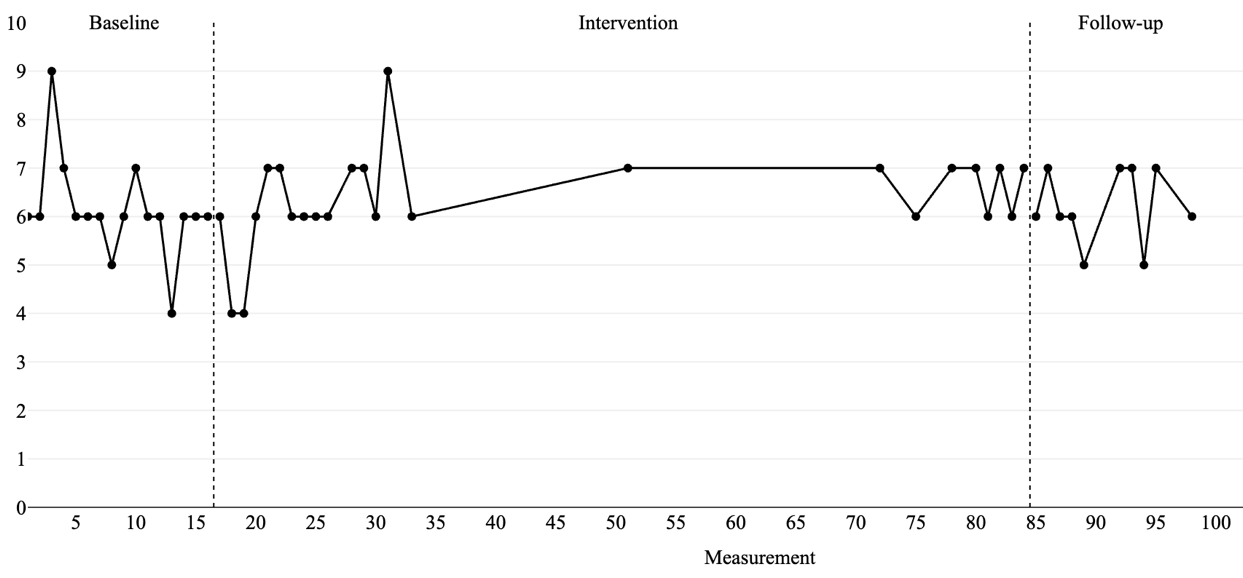
**Figure 4. Reflection, comprehense (reappraisal)**Item 4. In response to my emotions, I thought of other ways to interpret the situation – not at all (0) → very much (10)


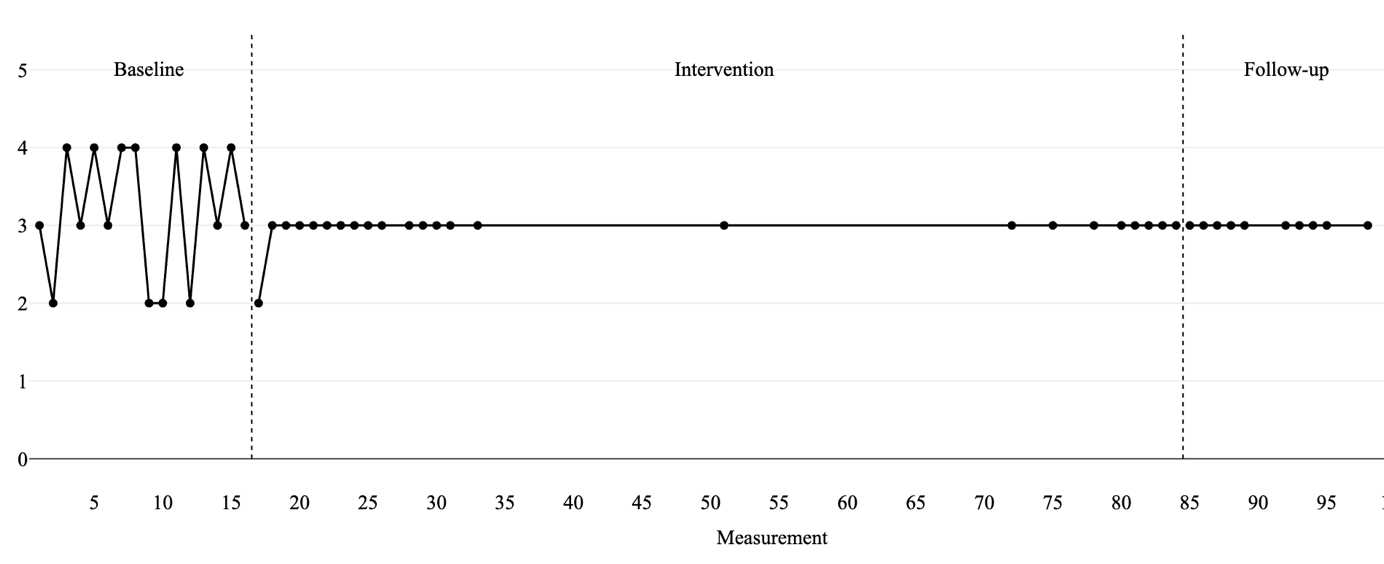
**Figure 5. Managing (impulse control)**Item 5. In the past 24 hours, when I'm upset, I had difficulty controlling my behaviors.- almost never (0) → almost always (5)

*Note. Item was reverse-coded, expected direction: increase*


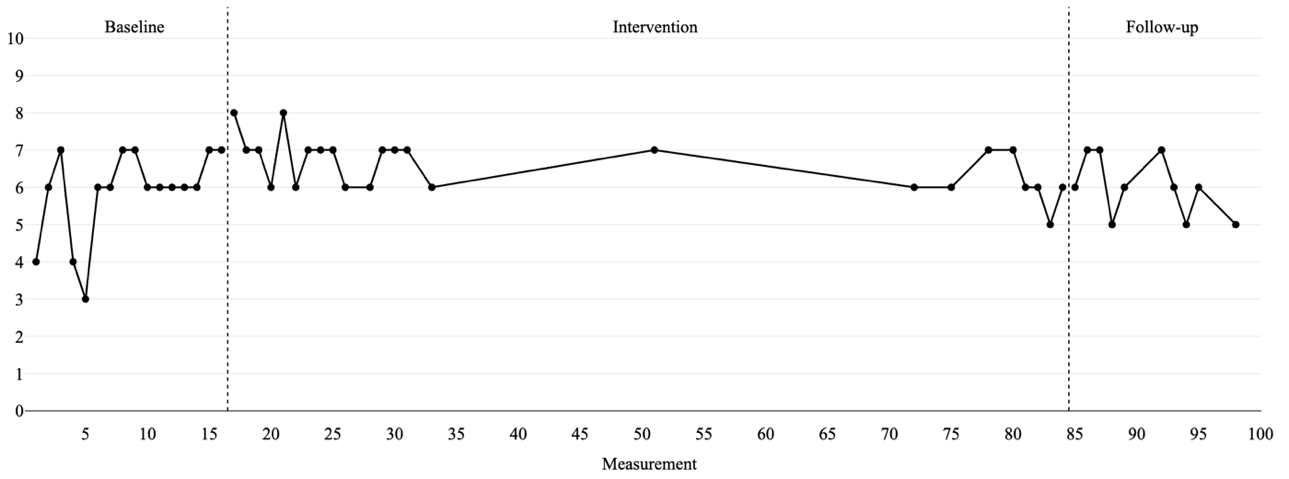
**Figure 6. Managing (distraction)**Item 6. In response to my emotions, I engaged in activities to distract myself – not at all (0) → very much (10)

**4. Secondary outcomes**

On secondary objectives, Adam’s RCI’s scores on the reflection (SRIS-Y) indicated a significant increase from T0-T1. However, the scores decreased between T1-T2 and T0-T2, and were found reliable. For self-insight (SRIS-Y), RCI-scores increased between T0-T1, but decreased between T1-T2. No reliable change could be found on scores between T0-T2. For emotional awareness, no reliable change on the MAIA could be noted. Last, for both treatment related outcomes, did Adam show no significant changes for both motivation (ATMQ) and alliance (WAV-12) scores throughout study.

**Tabel 1.** Results emotional factors

|  | **T0** | **T1** | **T2** | **RCI T0-T1** | **RCI T1-T2** | **RCI T0-T2** |
| --- | --- | --- | --- | --- | --- | --- |
| Positive affect (PANAS) | 18 | 22 | 26 | 1.84 | 1.84 | 3.68 |
| Negative affect (PANAS) | 17 | 17 | 16 | 0 | -0.42 | -0.42 |
| Self-reflection (SRIS-Y) | 45 | 48 | 39 | 1.33 | -3.98 | -2.65 |
| Insight (SRIS-Y) | 17 | 21 | 18 | 1.63 | 1.22 | 0.41 |
| Emotional awareness (MAIA) | 3 | 2,2 | 3 | -0.86 | 0.87 | 0 |

Note. RCI = significant at level < -1.96 or > 1.96.

**Tabel 2.** Results treatment factors

|  | **T0** | **T1** | **T2** | **RCI T0-T1** | **RCI T1-T2** | **RCI T0-T2** |
| --- | --- | --- | --- | --- | --- | --- |
| Treatment motivation (ATMQ) | 2,3 | 2,27 | 2,18 | -0.04 | -0.18 | -0.13 |
| Treatment alliance (WAV-12) | 49 | 55 | 57 | 1.97 | 2.62 | 0.66 |

Note. RCI = significant at level < -1.96 or > 1.96.

**5. Qualitative results**

In the interview, Adam noticed a pattern in his emotions throughout the week. He generally felt better towards the end of the week than at the beginning. He explained that this improvement was due to engaging in activities later in the week. At the beginning of the week, he tended to reflect more on why he felt bad and what he could do about it. As Adam explained:

*“A couple of weeks ago, I had a meeting at school that didn’t feel good. I logged my emotions, and later that week I looked back and realized why I had felt that way. It helped me understand my feelings and what I could do to change them.”*

Furthermore, Adam indicates that Feelee helps to think about emotions, something he often forgets:

*“It helped me remember my emotions, the setting, and what I was doing—so I could see how my feelings were connected to my activities".*

The clinician recognized this pattern and said it allowed them to explore emotions and situations in much more depth during treatment.

*“That was really helpful for us, (…) We could then look at the app together, and he could tell us who he was with and where he had been. (..) And that would often lead to a meaningful conversation with us.”* (C5).

Furthermore, the clinician stated that Feelee played a role in helping him feel better:

*“But I do think the Feelee app was part of why he started feeling better, because of the structure. It actually became part of his routine for a while.”*(C5).

Both acknowledged that, for Adam, Feelee contributed to a breakthrough in his treatment.
