# Supplementary material for: ‘I gotta Feeling’: Exploring the effects of a smartphone app (Feelee) to enhance adolescents’ emotion regulation in forensic outpatient settings: A multiple single-case experimental design
Source: PLoS One. 2026 Feb 6;21(2):e0332111. doi: 10.1371/journal.pone.0332111 (PMC12880710; doi:10.1371/journal.pone.0332111)
Supplement: Attachment — Submitted filename: Response to Reviewers.docx [file pone.0332111.s007.docx]

**Response to Reviewers**

We thank the editor and the reviewers for their constructive and insightful comments on our manuscript. Reviewer comments are presented below, followed by our responses. Changes made to the manuscript are indicated where relevant.

**Review Comments:**

**Reviewer #1:**

**Comment 1:** Authors must provide justification or rationale for choosing a 2-week baseline (A1), a 4-week intervention (B), and a 2-week follow-up phase (A2). What informed the duration of the intervention period, and what would have been the implications if the intervention period had been extended or reduced? Kindly provide justification, which may include references to similar studies, etc.

**Response comment 1**: We appreciate the reviewer’s comment regarding further justification for the chosen phase lengths. This point indeed reflects the key dilemma of the current study. On the one hand, we aimed to obtain sufficiently valid data according to SCED methodological guidelines, which recommend a minimum of three data points per phase to capture individual changes over time (Kazdin, 2021; Krasny-Pacini & Evans, 2018). On the other hand, we worked with a highly complex group of adolescents in forensic outpatient care, who are often difficult to include and retain over longer research periods. Extending the phases could have strengthened internal validity, but would likely have increased dropout and reduced data quality. Therefore, the chosen phase lengths (2-week baseline, 4-week intervention, 2-week follow-up) represent a careful balance between methodological rigor and feasibility, ensuring sufficient repeated measures while minimizing participant burden. We incorporated a brief explanation of this point on page 9.

**Comment 2**: The authors stated that three months after the initial follow-up measurement, a long-term follow-up assessment (T3) was conducted to evaluate the potential sustained effects of Feelee. What do you define as long-term? 6 months, 1 year, etc. Kindly clarify in the main document.

**Response comment 2**: We thank the reviewer for this observation. In the current study, long-term follow-up refers to the assessment conducted three months after the initial post-measurement (T2). We have clarified this definition in the methods section on page 9 to avoid ambiguity.

**Comment 3**: What informed the selection of participants from outpatient forensic teams at two mental healthcare institutions in the Netherlands? Are they the only mental health institutions in the Netherlands? What informed the author's choice of selecting Levvel and Inforsa and not any other mental health facilities? How did you arrive at only two facilities and not any other number? No power analysis? Clarity must be provided in the manuscript.

**Response comment 3**: We agree that clarification on the selection of participants and research sites is important. The present study was exploratory in nature, aiming primarily to gain initial insights into the potential effects of the intervention in a forensic outpatient population, rather than to statistically demonstrate causal effectiveness. For that reason, no a priori power analysis was conducted; the focus was on gathering rich, practice-based data to inform future larger-scale research. Participants were recruited from two outpatient forensic teams at Levvel and Inforsa, two established mental healthcare institutions in the Netherlands with outpatient forensic services. The selection of these sites was based on accessibility and existing collaboration with the research team, which enabled the researcher to obtain consent and organizational support for conducting the study. Inclusion of participants in this setting is generally challenging due to the complex nature of forensic outpatient care and the need for ethical and logistical approvals. Although only two institutions participated, both are representative of Dutch forensic outpatient services, providing treatment to a comparable population and using standard evidence-based forensic care models. The study therefore provides a reasonable reflection of this clinical context, while remaining feasible within the exploratory scope of the project. A short clarification of these considerations has been added to the methods section (pages 9–10) of the revised manuscript.

**Comment 4**: Kindly explain what you mean by “0–10” scale through linear rescaling” in the manuscript.

**Response comment 4**: We appreciate the reviewer’s request for clarification. The term “linear rescaling” refers to the proportional transformation of the original 5-point Likert scale (1–5) to a 0–10 scale to enhance interpretability and comparability across items. Specifically, each response value was multiplied by 2.5, resulting in an equivalent scale for all items ranging from 0 to 10. We have added this clarification to the methods section (page 16) of the revised manuscript.

**Comment 5**: Yes, the permutation test does not correct for autocorrelation, but how did you assume that the scores on the six daily items do not exhibit substantial autoregression without any rigorous statistical test? Additionally, repeated responses in this case scenario are naturally autocorrelated and must be accounted for in the statistical analyses.

**Comment 6**: I am wondering how applying a correction for autocorrelation could therefore result in spurious effects. The autocorrelation problem affects standard error estimation and the corresponding p-value estimates. Can you show the results, both autocorrelation corrected and uncorrected, as part of the sensitivity analysis?

**Response comment 5 & 6**: We appreciate this important point. Initially, we chose not to correct for autocorrelation because the daily measurements did not show substantial autoregression, and applying such a correction could introduce spurious effects. After reconsidering this point, we do agree with the reviewer and decided to apply the autocorrelation correction. Therefore, we (i) computed and reported lag-1 autocorrelations for each participant and item (see Supplementary File 2), and (ii) added Permutation Distance Test results for median differences between phases (new Table 3). This adjustment affected several results. This adjustment affected several results. We are convinced, however, that it allows readers to interpret phase effects more appropriately in relation to the observed serial dependence.

**Comment 7**: Kindly label the y-axis of the box-whisker plot

**Response comment 7:** We thank the reviewer for this suggestion. We have now labelled the y-axis to clearly indicate the observed scores presented in figure 4. We hope this clarification adequately addresses the reviewer’s comment.

**Comment 8:** The primary and secondary outcome measures have been clearly defined, and the authors included a measurement scale for each outcome measure. The intervention has been well described in the manuscript. The results are valid as they followed a standard statistical analytic approach

**Response comment 8**: We thank the reviewer for the positive evaluation of our description of the outcome measures, the intervention, and the analytic approach. We are pleased that these aspects were considered clear and appropriate.

**Reviewer #2:**

**Comment 1:** The introduction would benefit from a more comprehensive review of the current state of the art. The statement that “current interventions in forensic outpatient settings primarily aim to address the multiproblem profile by focusing on reducing risk factors and increasing protective factors” lacks sufficient context. It would strengthen the introduction to elaborate on what these current interventions typically entail, including examples of evidence-based or widely adopted practices.

**Response comment 1:** In response to this comment, we substantially revised the introduction to provide a more comprehensive overview of the current state of the art in forensic outpatient care. We now elaborate on page 3-4 what commonly used interventions entail, guided by the principles of the Risk-Need-Responsivity framework (Andrews et al., 2006; Bonta et al., 2007), and include examples of evidence-based and widely implemented approaches such as cognitive-behavioral interventions and family-based modalities. We believe that the revised introduction more accurately reflects the existing literature and addresses the reviewer’s suggestion.

**Comment 2:** To strengthen the rationale for the Feelee app, a more detailed review of existing digital mental health tools for adolescents is needed, particularly those incorporating ecological momentary assessment (EMA) or sensor-based monitoring. This would better situate the current study within the broader landscape of digital health innovations and help justify the unique contribution of Feelee in this setting.

**Response comment 2:** Together with the revisions made in response to the previous comment, we substantially updated the introduction to provide a more detailed and coherent rationale for the Feelee app. We specifically added an overview of the current literature on the use of digital mental health tools for adolescents in forensic outpatient care (pages 5–6). This addition provides a clearer understanding of the relevance of examining the added value of the Feelee app within this clinical setting.

**Comment 3:** The study includes participants aged 12 to 23. The manuscript should provide a rationale for this broad age range and clarify how developmental differences were accounted for in the analysis. Were any age-related differences observed in engagement or outcomes? If so, these should be explicitly reported.

**Response comment 3:** We understand the reviewer’s comment regarding the rationale for the chosen age range. We made adjustments in the first paragraph of the introduction to clarify why adolescents and emerging adults aged 12 to 23 were included. As described, this age range reflects the population typically seen in forensic outpatient services and aligns with the broader developmental period of adolescence and emerging adulthood. With regard to potential age-related differences, the present SCED design and limited sample size do not support reliable subgroup analyses or inferences about developmental variation in engagement or treatment effects. Although participants differ in age, they share the common clinical context of receiving specialized forensic outpatient care, and the study was not powered or designed to examine age-specific trajectories. We therefore did not conduct inferential analyses by age. We agree that investigating potential developmental differences in response to app-supported interventions is an important avenue for future research and have added this point to the discussion.

**Comment 4:** Some context regarding smartphone ownership, access, and usage habits among this population would help support the feasibility and relevance of the intervention. Is there existing data on smartphone use within forensic adolescent population and were there any barriers to technology use observed during the study?

**Response comment 4:** As described in the introduction on page 6, current review studies (Aarts et al., 2025; Kip et al., 2018) highlight a strong need and clear opportunities for mobile health tools, while also showing that research specifically focused on adolescents in forensic care is still very limited. Although precise smartphone-use statistics for forensic outpatient populations are scarce, data from the general adolescent population in the Netherlands indicate that smartphone ownership is nearly universal, with 99% of adolescents aged 12 and above owning a smartphone (CBS, 2020). Given that our study involves ambulatory forensic care, these figures are highly relevant for estimating feasibility. Within our own study, we observed no barriers related to smartphone ownership or basic device access. All participants were able to use the app on their own smartphones throughout the study period.

**Comment 5:** The mention of the Feelee app in the introduction appears premature and somewhat disconnected from the rest of the introduction. Its inclusion would be more appropriate in the discussion section, where its relevance can be evaluated in light of the findings.

**Response comment 5:** We agree with the reviewer’s comment. In revising the introduction, we removed the mention of the Feelee app throughout the introduction and rewrote the section to focus more strongly on the rationale derived from existing studies and current evidence in the field.

**Comment 6:** While it is noted that passive sensor data (e.g., sleep and number of steps) are collected via the Feelee app, a comprehensive list of all data types gathered passively should be provided and the advantages of collecting such data.

**Response comment 6:** We appreciate this sharp observation. The previous phrasing may have suggested that Feelee collects a broader range of passive data than is actually the case. On pages 12–13 we clarified that Feelee collects only two types of passive data: daily step count and hours of sleep.

**Comment 7:** The term "active data" may be confusing in the context of smartphone and sensor-based research. It may be clearer and more accurate to refer to this as "self-report data" to distinguish it from passively collected information

**Response comment 7:** The term active data is commonly used in the digital mental health and digital phenotyping literature (e.g., Insel 2017; Mohr et al., 2017; Onnela, 2021) to refer specifically to data generated through intentional user input. To avoid ambiguity in our manuscript, we have clarified this terminology by explicitly stating that, in the context of this study, active data refer to self-reported data provided by adolescents (e.g., EMA/ESM entries and questionnaires). These clarifications have been incorporated into the revised text on page 4.

**Comment 8:** The inclusion of "basic proficiency in smartphone use" as an eligibility criterion warrants clarification. Please explain how this proficiency was assessed, by whom, and whether any standardised or informal measures were used.

**Response comment 8:** Thank you for this helpful comment. We agree that this eligibility criterion benefits from clarification. The requirement to include “basic proficiency in smartphone use” was included at the request of the Medical Ethics Committee. As described in the manuscript, participant screening was conducted collaboratively by the research team and the referring clinician. During this brief screening, clinicians informally verified that the adolescent could perform simple smartphone actions (e.g., opening and navigating mobile apps). A formal test was not deemed necessary, given that smartphone use is nearly universal in this age group, with 99% of adolescents aged 12 and above in the Netherlands owning a smartphone (CBS, 2020). In practice, no participants experienced difficulties using a smartphone, and no questions or concerns regarding smartphone use were raised during screening or participation. A short clarification has been added to the manuscript on page 10.

**Comment 9:** More detailed information is needed regarding participant compensation. How much were participants compensated? The phrase “agreed frequency of updates” is unclear. Please clarify how and when participants were compensated and whether reminders or incentives were provided.

**Response comment 9:** Thank you for this comment. Participants received a fixed financial compensation for each completed daily questionnaire, each extended assessment (T0, T1, T2, and T3), and each emotion entry in the Feelee app. Participants who completed all daily questionnaires and submitted at least one emoji entry per day within a week received a small weekly bonus. The total possible compensation amounted to a maximum of €38, provided in the form of a gift voucher. At the start of the study (T0), the researcher and participant agreed on the preferred frequency of reminders about study activities and accumulated compensation. Participants could choose between one or several reminders per week; in most cases, a single weekly reminder was preferred. We added this additional information on page 11 of the manuscript.

**Comment 10:** Further information is needed on how data summaries from the Feelee app were generated and shared with participants’ and clinicians. It would be important to outline what guidance or training, if any, was provided to clinicians to support the integration of Feelee app data into their sessions with participants to provide context to its adoption and discussion of data with adolescents.

**Response comment 10:** Thank you for raising this point. We agree that additional clarification was needed on how Feelee data summaries were generated and discussed during treatment. The Feelee app automatically generated a dashboard that combined adolescents’ self-reported entries with passively collected sleep and step data. These dashboard overviews were reviewed jointly by adolescents and clinicians during the weekly treatment-as-usual sessions. To support clinicians in integrating these data into their therapeutic work, they received a two-part training prior to study start and a brief written handout summarizing the key steps and guidance. This additional information has been added on page 13 of the manuscript.

**Comment 11:** More information is needed about the qualitative component of the study. Specifically, an overview of the interview topic guide would be valuable. This should include the main domains or questions covered in the interviews and the analysis approach, to allow readers to assess the breadth and focus of the qualitative component of the study.

**Response comments 11:** This is a helpful observation from the reviewer. To provide a clearer overview of the qualitative component, we have added the full interview topic lists as Supplementary File 1. This appendix includes the main domains and questions covered in the interviews. In addition, we elaborated on the analytic approach in the Methods section to allow readers to better assess the breadth and focus of the qualitative component.

**Comment 12:** The themes presented are quite limited and are very categorical. The authors should distinguish between themes that were pre-defined and those that emerged through inductive thematic analysis. Clarify the approach to coding and provide illustrative quotes for each major theme.

**Response comments 12:** Thank you for this important comment. We have expanded the qualitative Methods section to provide a clearer explanation of the reflexive thematic analysis approach. The revised text on page 18 now distinguishes between the predefined domains from the topic guide (used as sensitizing concepts rather than fixed themes) and the inductively emerging patterns from the first coding round. We also clarify how these codes were discussed within the research team in a reflexive dialogue and how they were incorporated into the themes. These overarching themes are supported by subthemes, which are illustrated with quotes from adolescents and clinicians. We hope this addition further clarifies the analytic process and the structure of the qualitative findings.

**Comment 13:** How many qualitative interviews were conducted?

**Response comment 13:** Thanks for this question. A total of 31 qualitative interviews were conducted and analyzed. This information has been added on page 27 of the manuscript.

**Comment 14:** In the qualitative results section, the term ‘participants’ is used. As there are two participant groups (adolescents and clinicians) it would be advantageous to be explicit as to which group are being referred to.

**Response comment 14:** We agreed that using the general term participants created ambiguity given the two distinct groups involved. The qualitative results section has therefore been revised by specifying adolescents or clinicians in each instance, ensuring that it is clear which group is being referred to. These clarifications are included in the revised manuscript.

**Comment 15:** In the results it would be beneficial to separate out how many had their data discussed once and not at all rather than grouping these.

**Response comment 15**: We adjusted the results section to report separately how many adolescents had their Feelee data discussed once and how many did not have their data discussed at all. This information is now presented explicitly on page 21, with additional participant-level detail provided in Supplementary File 2.

**Comment 16**: Limited discussion of the data is reported. Did participants report any reasons for why it was not discussed?

**Response comment 16**: Thank you for this important comment. Adolescents mainly observed whether the Feelee data had been discussed, while clinicians provided more concrete explanations for why this did not always occur. During the initial reflexive coding process, this aspect did not emerge as a distinct subtheme. In response to the feedback received from multiple reviewers, and recognizing the importance of placing more emphasis on this point, we revisited this topic during the revision phase. In line with our reflexive analytic approach, the research team discussed the topic again, which led to the identification of this element as a new subtheme. We have therefore added this subtheme to the revised Results section on pages 30–31.

**Comment 17**: The presentation of the qualitative findings is also very limited and lacks details. The qualitative findings reference issues with passive data collection, but lack specificity. What types of issues were encountered? Providing adolescent or clinician quotes would help illustrate these challenges.

**Response comment 17:** Thank you for this helpful comment. This point reflects a dilemma we also encountered, namely how to include the qualitative findings as an addition to the quantitative results while keeping the manuscript concise. For reasons of length, we initially chose to present the qualitative results in a more limited way. We agree that this part needed more detail, especially regarding the issues with passive data collection. In the revised Results section, we therefore describe the specific problems adolescents and clinicians reported (for example inaccurate sleep estimates, data not working at all). We also added quotes from both adolescents and clinicians to illustrate these challenges more clearly. We hope these additions provide a clearer and more concrete picture of the qualitative findings without overwhelming the reader.

**Comment 18**: The paper could do we further proofreading as grammatical and spelling errors were identified (e.g. missing full stops, hypnotised should be hypothesised).

**Response comment 18**: Thank you for pointing this out. We reviewed the full manuscript and corrected the grammatical and spelling errors noted by the reviewer, including the examples mentioned. Additional proofreading has been conducted throughout the text.

**Comment 19**: Formatting should be reviewed, particularly the small, disjointed line beneath Table 1.

**Response comment 19**: This is a helpful remark from the reviewer. The formatting issue beneath Table 1 has been corrected. The layout of the table and surrounding text has been reviewed to ensure consistency.

**Comment 20**: Consider rephrasing “these multi problems” for clarity

**Response comment 20**: We revised the introduction, and in doing so rephrased this section to improve clarity. The wording has been adjusted and the phrasing “these multi problems” no longer appears in the revised version.

**Comment 21**: Some more sub-headings and signposting may improve the readability of the results.

**Response comment 21**: This is a helpful observation from the reviewer. To improve clarity and strengthen the structure of the Results section, we added additional sub-headings and clearer signposting in both the primary (quantitative) results and the qualitative findings. We hope that these adjustments make the progression of the results easier to follow and help highlight the main components of each section.

**Reviewer #3:**

Comment 1: Efficacy or effectiveness: while it is true that the authors carry out their pilot study in an outpatient setting involving two clinics, the use of the concept of effectiveness in this preliminary and research setting seems not appropriate.

**Response comment 1:** We agree that the term effectiveness may imply a level of experimental control and causal inference that does not apply to the present exploratory study. Our intention was not to claim causal effectiveness, but rather to examine whether initial changes in emotion regulation could be observed following the use of Feelee within routine outpatient care. To avoid misunderstanding, we have revised the terminology throughout the manuscript, replacing effectiveness with initial effects or preliminary findings where appropriate.

**Comment 2:** Explain the rationale of the timeline of the SCED timeline, why 2-weeks baseline, then 4 weeks intervention and then 2-weeks follow up phase? This seems to be very little time regarding the attempt to influence basic mechanisms such as emotion regulation training.

**Response 2:** We appreciate the reviewer’s comment regarding the rationale for the SCED timeline. While we acknowledge that deeper changes in emotion regulation develop over longer periods, the present study focused on initial changes associated with Feelee use across different components of emotion regulation. The study aimed to better understand how Feelee supports various steps of the emotion regulation process, and to explore which aspects may already show improvement and which remain challenging. The chosen durations (2-week baseline, 4-week intervention, 2-week follow-up) align with SCED methodological guidelines recommending at least 3 data points per phase (Kazdin, 2021; Krasny-Pacini & Evans, 2018), while maintaining feasibility within this complex forensic outpatient context. Extending the phases could have increased internal validity but would likely have reduced participant engagement. A brief clarification of this rationale has been added to the Methods section (page 9) of the revised manuscript.

**Comment 3:** Exchange the term “psychiatric” conditions with mental disorders, as “psychiatric” describes a profession and mental disorders describe the content of interest.

**Response 3:** We thank the reviewer for this helpful remark. The term psychiatric conditions has been replaced with mental disorders on page 10 of the revised manuscript.

**Comment 4**: Were comorbid mental disorders assessed? Please report them.

**Response 4**: We thank the reviewer for this comment. Comorbid mental disorders were not assessed in the present study. Only diagnostic information was collected, primarily indicating the focus and type of treatment, and this information is presented on page 19 of the manuscript.

**Comment 5**: Participants received monetary incentives: on what grounds did you choose this approach and how do you foster intrinsic motivation to continue using the techniques of the App?

**Response 5**: Thank you for this thoughtful question. The monetary incentive was provided to compensate adolescents for the time and effort required to complete the research procedures, particularly the daily app use and interviews. This approach is commonly used in EMA-based studies with adolescents to reduce study burden and support adherence during the data-collection period (Heron et al., 2017; Wen et al., 2017). Importantly, the incentive was not linked to clinical use of the app or to specific outcomes. Intrinsic motivation to apply the techniques offered by the app was fostered through the therapeutic context rather than through the incentive. Clinicians encouraged adolescents to use the app as part of their regular sessions, focusing on how the Feelee data could help them recognize emotions, reflect on daily patterns and support treatment goals. In this way, the financial compensation served only to support participation in the research procedures, while motivation to use the app’s techniques was embedded in the treatment process itself. However, we agree that this is an important point to acknowledge. A brief clarification has therefore been added in the method section on page 11-12.

**Comment 6**: Steps and “sleep” were assessed. Please indicate more clearly what the goal of this assessment was. Did you really expect that the short-term emotion regulation training would improve sleep and increase physical activity?

**Response 6:** We thank the reviewer for this relevant comment. Sleep and step count were assessed because these parameters are integrated features of the Feelee app and are discussed with adolescents as part of the therapeutic process. The purpose was not to evaluate improvements in sleep or physical activity, but to provide insights that could be reflected upon in therapy and support understanding of how daily routines relate to emotional functioning. We have clarified this point in the methods section (page 12) of the revised manuscript.

**Comment 7**: Add examples of the items of DERS and RESS-EMA, and indicate ICCs

**Response 7**: We appreciate this comment. The items used in the daily emotion-regulation measure were based on the original DERS-36 and RESS-EMA scales, but were not taken over one-to-one; instead, they were adapted for daily assessment by shortening the wording and adding a 24-hour reference frame. To clarify this process, we have included example items for each domain (recognition, reflection, and management) and described how the original items were transformed. This information has been added to page 14 of the manuscript. Furthermore, we appreciate the reviewer’s suggestion to report intraclass correlations (ICCs). However, in the context of the present Single-Case Experimental Design (SCED) with only a small number of participants and many repeated assessments per individual, ICC estimation is not meaningful or psychometrically informative. ICCs are designed to quantify the proportion of variance attributable to between-person differences relative to within-person variability. In SCEDs, however, the analytic focus is inherently intra-individual: each participant serves as their own control, and the primary goal is to model within-person change across phases. Because the number of participants is extremely limited and the between-person variance is therefore not reliably estimable, ICCs become unstable, biased, and uninterpretable in such small-N designs. Conversely, the within-person variance is well captured by the repeated measurements and directly incorporated into the phase-based SCED analyses. For these reasons, ICCs are typically not reported in SCED studies and would not contribute meaningfully to the interpretation of the present data. We have therefore not included ICC estimates.

**Comment 8:** On p. 15, you write that the permutation test, which does not correct for autocorrelation as it is assumed that the six daily items do not exhibit autoregression. This does not seem to be plausible.

**Response 8**: Thank you for raising this important point. Initially, we chose not to correct for autocorrelation because the daily measurements did not show substantial autoregression and applying such a correction could introduce spurious effects. After reconsidering this point, we decided to apply the autocorrelation correction. Therefore, we calculated lag-1 autocorrelations for each participant and item and report these values in Supplementary File 2. We also clarified the description of the permutation test to make explicit that this method does not adjust for serial dependence. To support a careful interpretation of the phase comparisons, we now present the permutation test results together with the median differences between phases (new Table 3), allowing readers to interpret these outcomes in light of the observed autocorrelation in each series. Finally, we added a brief note discussing how autocorrelation can affect significance estimates and why, in the context of our exploratory SCED approach, median shifts remain the primary indicator of within-person change.

**Comment 9**: Please explain why the three cases were chosen to be presented.

**Response 9**: We thank the reviewer for this comment. The three cases were selected to illustrate the range of individual trajectories observed in the study, representing distinct patterns of Feelee usage and change over time. A brief explanation of this selection has been added on page 18 of the revised manuscript.

**Comment 10**: Conclusions on the efficacy of the App are overstated. The findings may indicate the way for future research, including larger sample sizes and controlled designs, but interpretation of e.g. distraction values seems a bit too far-reaching. The same holds regarding the interpretation of the secondary effects and the delayed improvement of emotional differentiation and competencies (p.31). On the other hand, treatment integrity was very low, and this is a major issue that should be discussed in more detail. Why did therapists not engage? Is this a common problem in this patient-therapist group?

**Response 10**: Thank you for this helpful comment. We agree that the original discussion overstated the conclusions. In the revised manuscript, we have substantially toned down the interpretation of both the primary and secondary findings. All effects are now framed as exploratory within-person patterns, without implying efficacy or causal influence. Interpretations of items such as distraction and the secondary outcomes have been rewritten to reflect alternative explanations and measurement limitations. We also expanded the section on treatment integrity. The qualitative data showed that clinicians often deprioritised the app because other issues in treatment required immediate attention. This is a well-known challenge in forensic outpatient care, where acute and complex needs frequently shift the focus of sessions. We now explicitly acknowledge this as a limitation that affects the interpretation of the findings. Taken together, these revisions ensure that the discussion is more cautious, balanced and transparent, and position the findings as preliminary signals that require confirmation in future research.

**Reviewer #4**

**Comment 1:** Firstly, the study adopts a multiple single-case experimental ABA design but lacks a well-defined control group, which limits the interpretability of the reported reduction in emotional suppression. It remains unclear whether the reduction in emotional suppression resulted from the Feelee app itself or from concurrent forensic outpatient treatment. Forensic outpatient care inherently includes therapeutic elements that may independently influence emotion regulation. Moreover, adolescents under judicial supervision may show behavioral improvement due to legal or compliance-related factors. As currently designed, the study cannot disentangle the effects of the app from those of the ongoing forensic outpatient program.

**Response comment 1:** We appreciate this thoughtful observation. Indeed, the current multiple single-case experimental (ABA) design does not include an independent control group. However, in Single-Case Experimental Designs (SCEDs), each participant effectively serves as their own control. This design substantially reduces between-subject variability and controls for many sources of within-person error, allowing meaningful evaluation of within-individual change across systematically introduced phases. We fully acknowledge that forensic outpatient treatment and contextual factors (e.g., judicial supervision, compliance motivation) may also contribute to changes in emotional suppression. This is inherent to the ecological, practice-based setting of the study. Yet, the visual inspection analyses clearly show substantial within-person variability during baseline and follow-up phases, with a distinct phase-specific change coinciding with the introduction of the Feelee app. Therefore, any observed intervention effect must be interpreted as an effect above and beyond these naturally occurring or treatment-related fluctuations. We have clarified this point in the Discussion section, emphasizing that while causal attribution cannot be fully separated from concurrent care, the single-case design provides a strong initial indication that observed changes are temporally associated with the intervention phase.

**Comment 2:** Secondly, the manuscript does not provide a rationale or statistical justification for the chosen sample size. No reference to prior effect sizes from studies on emotion suppression or app-based emotion regulation was presented. Although 27 adolescents were recruited, only 14 were included in the main analysis and 13 in the final dataset. The authors should clarify whether this number affords sufficient statistical power to detect the intended effects. Without such justification, the robustness of the reported findings remains uncertain.

**Response comment 2**: We appreciate the reviewer’s concern regarding statistical power. The present study was explicitly exploratory, aiming primarily to assess the initial effects of implementing the Feelee app within forensic outpatient care and to explore potential patterns of change in emotion regulation. The design was therefore not intended to test strong confirmatory hypotheses or to provide inferential proof of effectiveness. In this context, “power” should be understood more broadly as the capacity of the design to detect observable change if such change truly occurs within individuals. The multiple single-case experimental (ABA) structure, with repeated measurements per participant, provides substantial within-person replication and sensitivity to individual-level change, even with a modest number of cases. We acknowledge, however, that the findings should be viewed as preliminary and primarily informative for future confirmatory research. We have clarified this rationale and limitation in the revised manuscript.

**Comment 3**: Furthermore, the statistical analyses involve numerous comparisons across phases and variables, yet there is no indication that corrections for multiple testing (e.g., Bonferroni) were applied. In addition to these major concerns, the manuscript provides only minimal information about the nature of the forensic outpatient care provided to participants. It is unclear what kinds of offenses or behavioral problems characterized these adolescents, what psychiatric or psychological conditions they presented with, and what specific treatments (“treatment as usual”) they received. Details such as session content, therapeutic approaches, and treatment duration are essential for evaluating the study context and the plausibility of treatment effects.

**Response comment 3:** We appreciate the reviewer’s concern regarding multiple testing. However, the current study employs an idiographic Single-Case Experimental Design (SCED), in which inferential focus lies on within-person change across phases rather than on pooled or aggregated hypothesis testing. Because each participant serves as their own control, and because analyses are not used to draw population-level inferences across variables or participants, conventional corrections for multiple comparisons (e.g., Bonferroni) are not applicable. Applying such corrections would assume independent statistical tests aimed at estimating a single population parameter—an assumption incompatible with the logic and purpose of SCED methodology. Instead, the analyses reflect the expected variability in individual trajectories and phase-based effects across participants, which is central to idiographic research.

**Comment 4**: Lastly, the qualitative findings mainly describe user experiences with the app, rather than offering explanatory insight into how emotional suppression was reduced. The current qualitative results could be condensed, and integration of qualitative and quantitative findings—either within the discussion or in supplementary materials—would provide a more coherent interpretation of the app’s effects.

**Response comment 4**: Thank you for this helpful comment. We have revisited the qualitative section to ensure that its contribution is clearly articulated. While the qualitative findings included adolescents’ and clinicians’ experiences with using the app, they also offered insight into how participants experienced potential emotion-related effects. Furthermore, to provide a clearer integration, we have strengthened the links between these qualitative observations and the quantitative patterns in the revised discussion. In addition, we have condensed parts of the qualitative results to keep the focus on their explanatory value and to provide a more coherent integration of the findings.
