# Supplementary material for: ‘I gotta Feeling’: Exploring the effects of a smartphone app (Feelee) to enhance adolescents’ emotion regulation in forensic outpatient settings: A multiple single-case experimental design
Source: PLoS One. 2026 Feb 6;21(2):e0332111. doi: 10.1371/journal.pone.0332111 (PMC12880710; doi:10.1371/journal.pone.0332111)
Supplement: Attachment — Submitted filename: Response_to_Reviewers_auresp_2.docx [file pone.0332111.s008.docx]

**Response to Reviewers**

We thank the editor and the reviewers for taking the time to carefully re-evaluate our revised manuscript and our responses to the previous review round. We are pleased that the revisions and clarifications have addressed the reviewers’ earlier concerns. Below, we provide our responses to the remaining review comments. All changes have been incorporated into the revised manuscript, with page numbers indicated where relevant.

**Review Comments:**

**Reviewer #1:** The authors have comprehensively addressed all the comments and concerns raised in my previous review of the manuscript.

**Comment reviewer #1:** We thank the reviewer for their careful re-evaluation of the revised manuscript and for this positive assessment.

**Reviewer #4:** I thank the authors for their thoughtful responses and acknowledge both the value and rarity of the dataset, as well as the practical and ethical constraints of conducting research in a forensic outpatient setting. Considering these issues, I accept the authors’ clarifications regarding the limitations of causal inference in the single-case design, the exploratory nature of the study and its implications for sample size, and the scope of the qualitative analyses. However, I remain concerned that the response to the issue of multiple statistical comparisons does not adequately address the core methodological concern raised in my original review.

While the authors argue that conventional corrections for multiple testing (e.g., Bonferroni) are not applicable within an idiographic Single-Case Experimental Design (SCED) framework, this argument does not sufficiently resolve the underlying issue. The concern is not the specific choice of correction method per se, but rather the extensive number of inferential statistical comparisons conducted across phases and outcome variables, and the absence of a clearly articulated strategy to manage, mitigate, or transparently acknowledge the resulting inflation of Type I error risk.

Simply stating that Bonferroni-type corrections are incompatible with SCED methodology is insufficient when numerous statistical tests are nevertheless reported and interpreted. At present, the manuscript does not clarify how multiplicity is handled analytically, whether alternative safeguards (e.g., emphasis on visual analysis, effect size patterns, consistency across phases, or other SCED-appropriate approaches) are employed, or how readers should interpret statistical significance in light of this multiplicity.

I therefore strongly encourage the authors to revisit and re-analyze the data using an explicit and methodologically coherent strategy to address the issue of multiple comparisons, or to substantially revise the analytical framework and interpretation to ensure that the risk of false-positive findings is appropriately controlled or transparently contextualized within the logic of SCED research.

**Comment reviewer #4:** We acknowledge that the number of statistical comparisons reported across phases and outcome variables increases the nominal probability of Type I error when p-values are considered in isolation. Within the logic of idiographic Single-Case Experimental Designs (SCEDs), however, these analyses do not constitute a single family of hypothesis tests aimed at population-level inference. As stated in foundational SCED methodology (e.g., Kratochwill et al., 2013; Krasny-Pacini & Evans, 2018), each comparison pertains to a specific participant, outcome, and phase contrast, and is intended to support descriptive and exploratory inference rather than confirmatory statistical testing.

To address multiplicity in a methodologically coherent manner, statistical significance testing is therefore not interpreted as a primary decision criterion. Instead, inference is based on the convergence of multiple, SCED-appropriate sources of evidence, including: (a) systematic visual inspection of level, trend, and variability across phases; (b) non-overlap–based effect size estimates (TAU-U), which quantify the degree and direction of change between phases while accounting for baseline trend; and (c) consistency of effect patterns across phase contrasts and participants.

Within the manuscript, p-values are reported for transparency but are interpreted descriptively and in conjunction with visual and effect size evidence, rather than dichotomously. Readers are explicitly cautioned in the discussion (page 32) against interpreting isolated statistically significant findings as confirmatory evidence. Instead, the strength of inference is derived from the replication logic of SCEDs and the convergence of visual and quantitative indicators.
